# Effects of TRAF3 on the proliferation and migration of lung adenocarcinoma depend partly on pyroptosis

**DOI:** 10.1186/s12885-023-11468-z

**Published:** 2023-10-05

**Authors:** Wangjia Wang, Shiqi Wang, Min Wang, Yamei Ma, Wanting Hu, Binsha Wu, Chichi Li, Dan Zhang

**Affiliations:** 1 Department of Respiratory and Critical Care Medicine, The First Affiliated Hospital of Wenzhou Medical University, Wenzhou City, 325000 China; 2 Department of Rheumatism and Immunology, Shangyu People’s Hospital, Shaoxing, 312300 China; 3 Department of Plastic Surgery, The First Affiliated Hospital of Wenzhou Medical University, Wenzhou City, 325000 China

**Keywords:** Tumor necrosis factor receptor-associated factor 3 (TRAF3), Lung adenocarcinoma (LUAD), Pyroptosis

## Abstract

**Background:**

Tumor necrosis factor receptor-associated factor 3 (TRAF3) has specific regulatory effects on a wide range of diseases, including tumors. However, the effect and mechanism of TRAF3 on lung adenocarcinoma (LUAD) are still unknown. The aim of the present study was to make clear the role and potential mechanism of TRAF3 in LUAD.

**Methods:**

TIMER2.0 database and western blot were applied to detect the expression of TRAF3 in lung adenocarcinoma tissue. Kaplan-Meier Plotter database was utilized to explore the effect of TRAF3 on the clinical prognosis of lung adenocarcinoma patients. Specific siRNA was used to inhibit the expression of TRAF3 in LUAD cells (A549 and H1299). CCK-8 and EdU assays were performed for assessing LUAD cells proliferation. Wound healing assay and transwell assay were performed for determining cells migration. CCK-8 assay was used to assess the response of the LUAD cells to paclitaxel. TIMER2.0 bioinformatics and western blot were employed to detect the effects of TRAF3 on pyroptosis in LUAD.

**Results:**

TRAF3 was highly expressed in lung adenocarcinoma tissues and cell lines. Patients with TRAF3 hyperexpression had a good prognosis compared to those with lower expression. TRAF3 inhibition notably induced proliferation and migration of LUAD cells. Inhibition of TRAF3 also weakened the sensitivity of LUAD cells to paclitaxel. Moreover, bioinformatics results showed that TRAF3 was positively correlated with the expression of pyroptosis-related genes in LUAD. Western blot assays showed that TRAF3 inhibition visibly decreased the expression of apoptosis-associated speck-like protein (ASC), cleaved caspase-1 and matured- IL-1β.

**Conclusions:**

Inhibition of TRAF3 promotes the proliferation and migration of LUAD cells, and reduces the sensitivity of LUAD cells to paclitaxel. The effects of TRAF3 on LUAD cells were mediated in part by caspase-1-dependent pyroptosis.

**Supplementary Information:**

The online version contains supplementary material available at 10.1186/s12885-023-11468-z.

## Introduction

Lung cancer is one of the malignancies with a high death rate worldwide. Lung adenocarcinoma (LUAD) is the most prevalent subtype of lung cancer, accounting for approximately 50% of all confirmed lung cancer [1]. Due to the lack of specific symptoms in the early stage, the optimal treatment period is often missed and the available treatment options are very limited [2]. In recent years, advances in biomarkers and targeted therapies have altered the clinical management of lung cancer patients. The identification of epidermal growth factor receptor (EGFR) gene mutations and the therapeutic efficacy of EGFR tyrosine kinase inhibitors (EGFR-TKIs) in this subgroup of tumors established the basis for biomarker-driven lung cancer therapy [3]. More molecular targets such as anaplastic lymphoma kinase (ALK), proto-oncogene tyrosine-protein kinase 1 (ROS1) and v-raf murine sarcoma viral oncogene homolog B1 (BRAF) also emerged and have shown promising efficacy [4]. However, the biggest obstacle to targeted therapy is the inevitable emergence of drug resistance [5]. Therefore, it is urgent and important to find new therapeutic targets for oncology treatment.

Pyroptosis, a form of inflammatory cell death, is closely related to various human diseases, especially malignant tumors [6]. Caspase-1, a major effector protease of pyroptotic pathway, is recruited and activated by the caspase recruitment domain (CARD) of apoptosis-associated speck-like protein (ASC), promotes the maturation and release of inflammatory mediators such as IL-1β and IL-18, and then triggers pyroptosis of cancer cells [7]. Relevant studies have shown that some chemotherapeutic agents work by inducing cell pyroptosis, and pyroptosis related factors have important clinical value for the prognosis of cancer [8]. However, pyroptosis often has a dual effect. On the one hand, pyroptosis can improve immune activity and effectively remove cancer cells those are resistant to apoptosis or necrosis. On the other hand, excessive pyroptosis causes the release of numerous inflammatory factors, forming an inflammatory microenvironment suitable for the growth of tumor cells [9]. Further clarifying of the relationship between pyroptosis and tumors could help to open up new directions for tumor treatment.

Tumor necrosis factor receptor-related factor 3 (TRAF3) is a multifunctional intracellular signal molecule protein, which plays an important regulatory role in inflammation and immune response by participating in the formation of distinct protein complexes [10]. TRAF3 forms a ubiquitinated complex with TRAF2 and apoptosis proteins (cIAPs) to facilitate nuclear factor κB (NF-κB)-inducing kinase (NIK) degradation and negatively regulate NF-κB-related signaling [11]. TRAF3 also participates in the formation and release of the MEK kinase 1 (MEKK1) and TGF-β-activated kinase 1 (TAK1) complexes, so that to negatively regulate mitogen-activated protein kinase (MAPK) signaling [12]. In addition, TRAF3 is also involved in regulating the survival, biological functions and malignant mutations of B-lymphocytes and myeloid cells. Researchers have identified frequent deletions or mutations of TRAF3 in human B-cell malignancies, and found that B cell-specific deletion of TRAF3 in mice significantly raised the incidence of B-cell lymphoma [13]. Consistent with the above, myeloid cell-specific TRAF3-deficient mice undergo spontaneous inflammation and tumors by the age of 15 to 22 months [14]. TRAF3 has also been shown to be involved in the pathogenesis of other tumor types, including nasopharyngeal, breast, colon and liver cancers [15]. However, the role of TRAF3 in lung cancer and its molecular mechanisms remain unclear. In the present study, we assessed the function of TRAF3 in LUAD cells by downregulating TRAF3 expression. Our findings suggest that TRAF3 inhibition can facilitate the proliferation and migration of lung adenocarcinoma cells, and reduce the sensitivity of LUAD cells to paclitaxel, partly by mediating the caspase-1-dependent pyroptosis.

## Methods

### Cell culture and transfection

Human LUAD cell lines (A549, H1299) and human bronchial epithelial cell (16HBE) were obtained from Cell Bank of the Chinese Academy of Science (Shanghai, China). A549 and H1299 cells were cultured in DMEM medium (Gibco, USA), 16HBE cells were cultured in RPMI‑1640 medium (Gibco, USA). All culture media contained 10% FBS. All the cells were incubated in an incubator at 37 ˚C with 5% CO_2_. The siRNAs of TRAF3 (si-TRAF3: 5’-CGUGUCAAGAGAGCAUCGUUATT-3’, 3’- UAACGAUGCUCUCUUGACACGTT-5’) and negative control (si-NC) were purchased from GenePharma Co. Ltd (Shanghai, China). LipofectamineTM 3000 transfection reagent (Invitrogen, Carlsbad, CA, USA) was used to transfect siRNAs into A549 and H1299 cells, and cultured for 24–48 h for subsequent experiments.

### TIMER 2.0 database

Timer 2.0 (http://timer.cistrome.org/) is a public database that provides a platform to explore differences and correlations in gene expression among different cancer species. We operated the “Gene_DE” and “Gene_Corr” module to gain the differential expression of TRAF3 between LUAD and normal tissues, as well as its correlation with pyroptosis-related genes. The gene expression levels were displayed by log2 TPM and conducted by spearman analysis.

### Kaplan-meier plotter database

Kaplan-Meier Plotter (https://kmplot.com/analysis) is an online survival analysis tool. TRAF3 was input as Gene symbol, with the truncation value set as the median expression value, and lung adenocarcinoma was selected for analysis in the Histology column. The survival analysis results of LUAD patients with high and low TRAF3 expression were represented by risk ratio (HR), logrank P value, and 95% confidence intervals.

### CCK-8 proliferation assay

Transfected A549 and H1299 cells were seeded in 96-well plates with 3 × 10^3^ cells per well. 10 ul of CCK-8 reagent (MCE, USA) was added to each well at different time points (24, 48, 72, 96 h), and continually incubated for 2 h. The absorbance OD value at 450 nm was then measured with a multi-mode microplate reader (Molecular Devices, Shanghai, China), and the cell viability curve was further drawn.

### EdU proliferation assay

Resuspended transfected cells were seeded in 96-well plates, and cultured at a density of 2 × 10^4^ cells per well for 24 h. Cell EdU labeling, immobilization, purifying, EdU detecting and nuclear staining were carried out according to the instructions of the BeyoClickTM EdU Cell Proliferation Kit with Alexa Fluor 555 (Beyotime, China).

Cell proliferation was observed and analyzed by a high throughput microplate imager (Operetta CLS®™, PerkinElmer, Beijing, China).

### Scratch wound healing assay

The treated cells were cultured in 12-well plates until the cells were 90% confluent. A 200 ul pipette tip was used to scratched vertically across the cell surface. Subsequently, washed 3 times with PBS, and replaced with serum-free medium. At 0, 24, 48, and 72 h of the experiment, the cell photo was recorded with a high throughput microplate imager (Operetta CLS®™, PerkinElmer, Beijing, China), and the healing rate was calculated using Image J version 8 (https://imagej.nih.gov/ij/).

### Transwell migration assay

600 ul of 10% FBS-containing medium was added into the lower chamber of the Transwell (Corning, USA). Serum-free cell suspensions were added into the upper chamber of Transwell at a density of 1.5 × 10^4^ cells/well (200 ul) for cell migration experiments. After the cells were cultured for 24 h, fixed with 4% paraformaldehyde for 50 min, and then stained with crystal violet for 30 min. The upper chamber was wiped gently with a cotton swab and dried slightly. The 5 fields were randomly selected under the microscope (Nikon, Tokyo, Japan), and the number of transmembrane cells were calculated.

### Paclitaxel toxicity detection

Pretreated lung adenocarcinoma cells were seeded in 96-well plates at a density of 5 × 10^3^ cells/well. After incubating for 24 h, cells were adhered and grown in the wells. Then, 10 ul of paclitaxel (GlpBio, USA) was added at different concentrations and incubated for 48 h. The cell viability was measured by CCK-8 assays. The data were analyzed with GraphPad Prism 8 software and expressed as a percentage of cell viability relative to the control group.

### Western blot analysis

Total proteins of lung adenocarcinoma cells were extracted using ice-cold RIPA lysis buffer, separated by SDS-PAGE and transferred to polyvinylidene difluoride membranes. After blocking with protein-less rapid blocking fluid for 15 min, the membranes were then incubated overnight with primary antibodies listed as follows: TRAF3 (#4729, Cell Signaling Technology, Danvers, MA, USA), IL-1β(#12,703, Cell Signaling Technology), cleaved IL-1β(#83,186, Cell Signaling Technology), GAPDH (#5174, Cell Signaling Technology), Anti-pro Caspase-1 + p10 + p12 (ab179515, Cambridge, MA, USA), Anti-TMS1/ASC (ab151700, Cambridge). Subsequently, membranes were incubated with horseradish peroxidase-conjugated goat anti-rabbit immunoglobulin IgG for 2 h at room temperature. Finally, the relative band intensity was quantified using Image J software and normalized against GAPDH.

### Statistical analysis

Each experiment was completed independently at least 3 times with independent samples from different biological sources, and the results were expressed as mean ± standard deviation (SD). Data analysis was performed by GraphPad Prism 8.0 software (San Diego, CA, USA). One-way ANOVA was used to analyze intergroup differences, and the differences were considered statistically significant when P < 0.05.

## Results

### TRAF3 is upregulated in LUAD

**Fig. 1:**
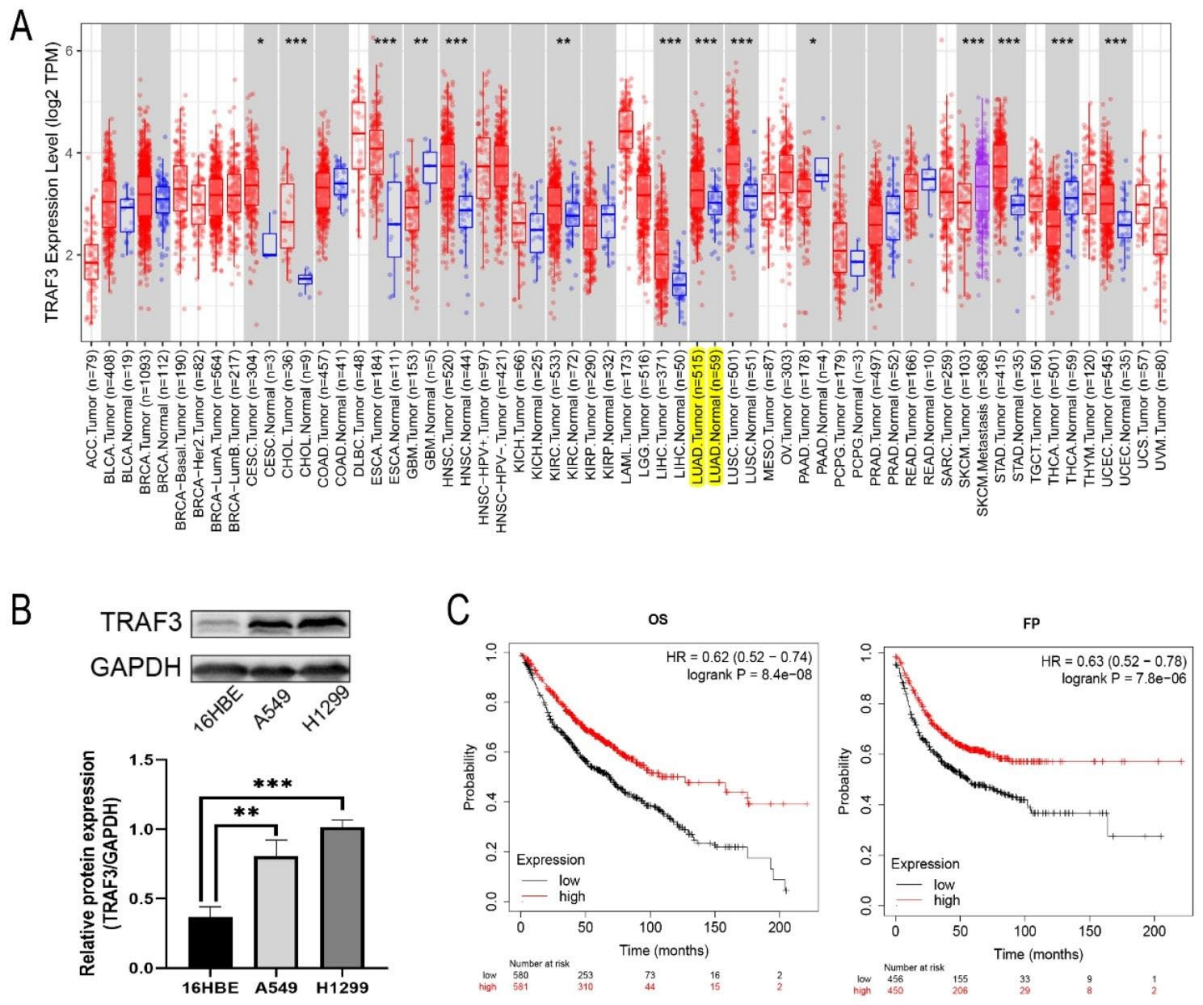
TRAF3 is upregulated in LUAD. **(A)** TIMER2.0 database showed the relative expression of TRAF3 in lung adenocarcinoma and adjacent tissues. *P<0.05, **P<0.01, *** *P*<0.001 versus normal tissues group. **(B)** Western blot assays revealed the relative expression of TRAF3 in human bronchial epithelial cells (16HBE) and human LUAD cell lines (A549, H1299). **P<0.01, ***P<0.001 versus 16HBE cell line. The results were presented as the mean ± SD (n = 3). **(C)** Kaplan-Meier Plotter database showed the relationship between TRAF3 expression levels with lung adenocarcinoma patients’ prognosis

In the TIMER2.0 database (http://timer.cistrome.org), we observed that the expression lever of TRAF3 in LUAD tissues was significantly higher than that in normal lung tissues (LUAD tissues = 515 cases, normal lung tissues = 59 cases, Fig. 1A). Subsequently, we detected TRAF3 in human bronchial epithelial cells (16HBE) and human LUAD cell lines (A549, H1299) by western blot. As shown in Fig. 1B, the protein expression level of TRAF3 was markedly upregulated in lung adenocarcinoma cells compared with that in normal lung epithelial cells (P<0.01). Next, we used the Kaplan-Meier Plotter to explore the effect of TRAF3 on the clinical prognosis of lung adenocarcinoma patients. Interestingly, the result revealed that high TRAF3 expression was associated with improved patients’ overall survival (OS, HR = 0.62, P<0.001, Fig. 1C) and first progressive (FP, HR = 0.63, P<0.001, Fig. 1C).

### Downregulation of TRAF3 promotes the proliferation of LUAD cells

**Fig. 2:**
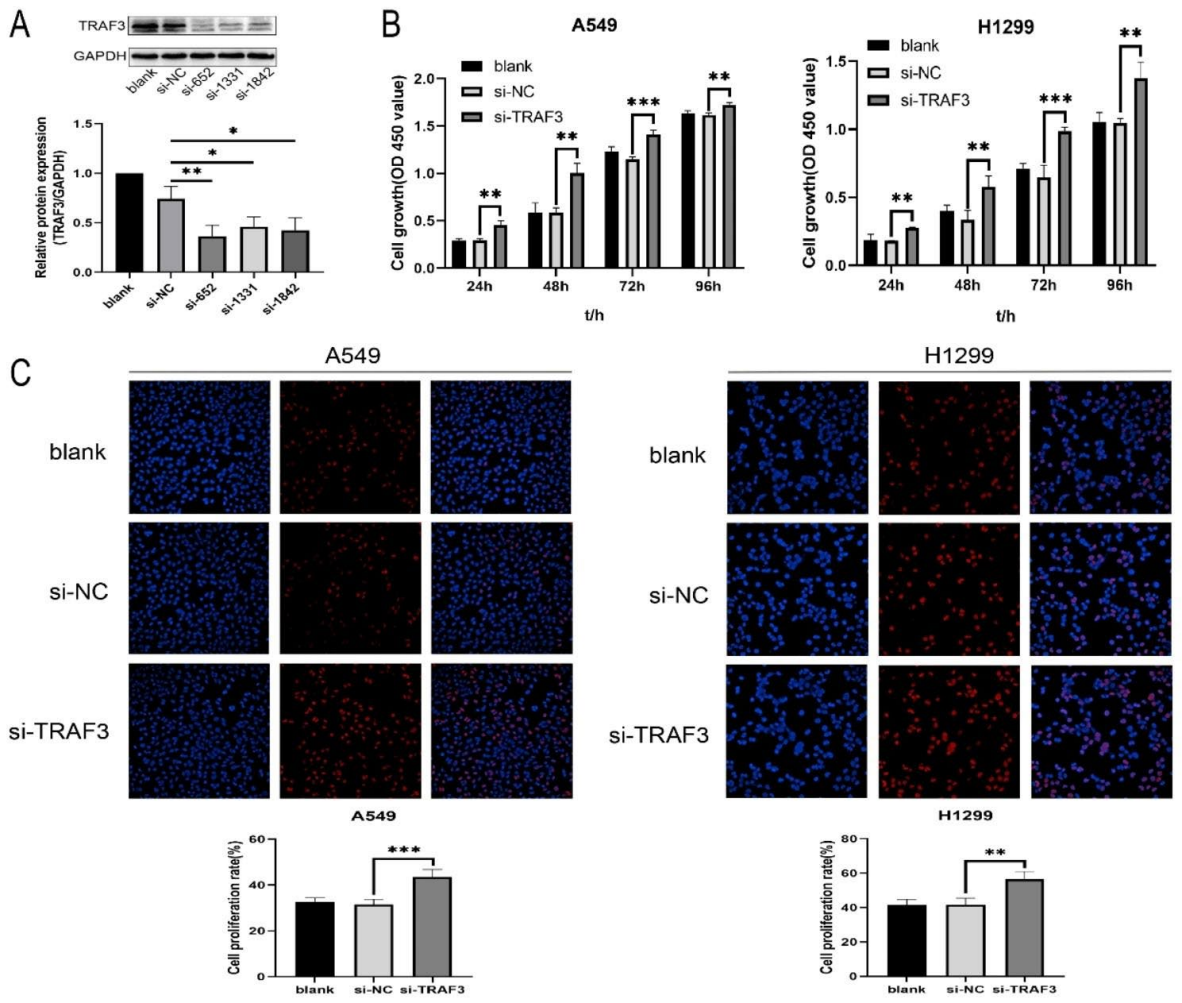
Downregulation of TRAF3 promotes the proliferation of LUAD cells. **(A)** Western blot was performed to evaluate the efficiency of si-*TRAF*3. *P<0.05, **P<0.01 versus control group. **(B)** CCK-8 assay was used to detect the proliferation activity of LUAD cells after transfection. **P<0.01, ***P<0.001 versus control group. **(C)** EdU assay was applied to determine the DNA replication ability of LUAD cells after transfection. **P<0.01, *** *P*<0.001 versus control group. The results were presented as the mean ± SD (n = 3–4)

To investigate the effects of TRAF3 on the biological behaviors of LUAD cells, negative control siRNA (si-NC) and specific siRNA targeted to TRAF3 (si-*TRAF*3) were transfected into A549 cells, respectively. It was showed that the expression of TRAF3 in si-*TRAF*3 treated group was significantly less than that in si-NC treated group (Fig. 2A). Subsequently, the siRNA with the most significant inhibitory effect on the expression level of TRAF3 was selected for follow-up experiments. CCK-8 and EdU assays were applied to explore the effect of TRAF3 downregulation on the proliferation of LUAD cells. The results revealed that knockdown of TRAF3 markedly promoted proliferation of A549 and H1299 cells (Fig. 2B, 2 C). As a result, TRAF3 performs an important role in modulating the proliferation of LUAD cells.

### Downregulation of TRAF3 promotes the migration of LUAD cells

**Fig. 3:**
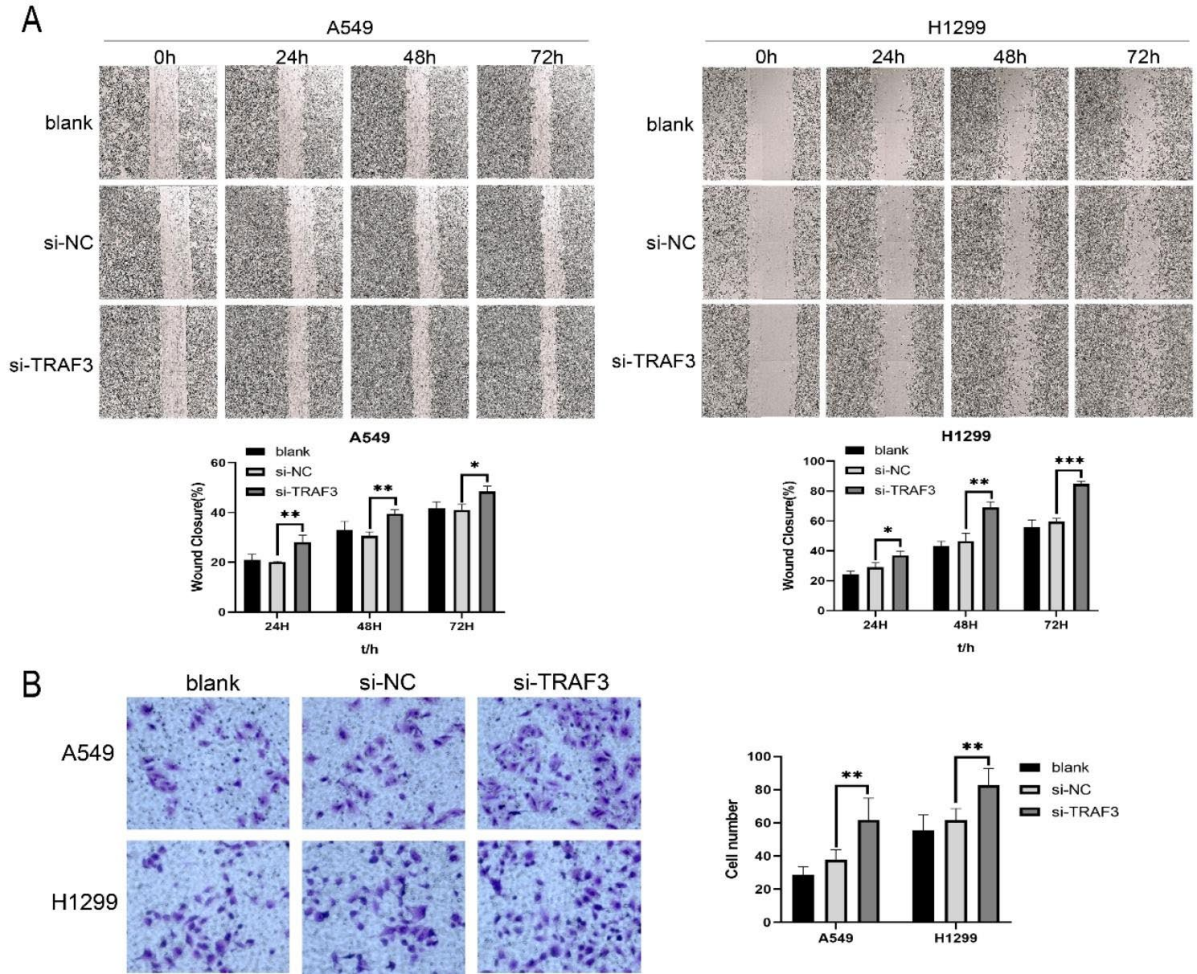
Downregulation of TRAF3 promotes the migration of LUAD cells. **(A)** Scratch test was used to detect the migration and healing of LUAD cells after transfection. **(B)** Transwell assay was used to detect the migration of LUAD cells after transfection. **P<0.01 versus control group. The results were presented as the mean ± SD (n = 3)

To assess the effect of TRAF3 on cell migration, scratch wound healing assay and transwell assay were performed. In the scratch experiment, knockdown of TRAF3 significantly promoted the migration ability of LUAD cells (Fig. 3A). These results were consistent with the transwell assay, in which migration activity was enhanced when TRAF3 was downregulated (Fig. 3B). Taken together, these results shown that downregulation of TRAF3 promotes the migration ability of LUAD cells.

### Downregulation of TRAF3 reduces the sensitivity of LUAD cells to paclitaxel

**Fig. 4:**
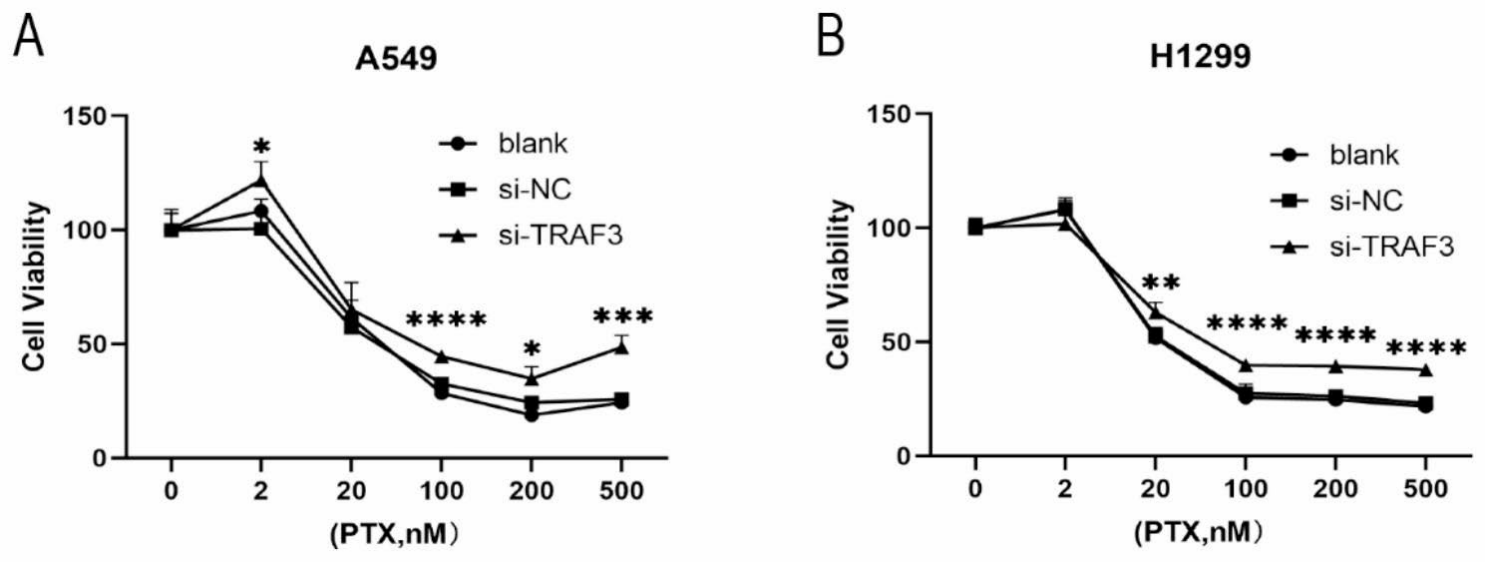
Downregulation of TRAF3 reduces the sensitivity of LUAD cells to paclitaxel **(A)** CCK-8 assay was applied to evaluate the effect of different concentrations of paclitaxel on the cell viability of A549 cells after transfection. *P<0.05, ***P<0.001, ****P<0.0001 versus control group. **(B)** CCK-8 assay was applied to evaluate the effect of different concentrations of paclitaxel on the cell viability of H1299 cells after transfection. **P<0.01, ****P<0.0001 versus control group. The results were presented as the mean ± SD (n = 3–6)

Paclitaxel (PTX) is a common first-line drug in chemotherapy, and has displayed good efficacy in the early stage of lung adenocarcinoma. However, the relatively rapid acquisition of resistance to PTX therapy significantly restricts its practicability. In this study, we treated LUAD cells with low expression of TRAF3 with different concentrations of paclitaxel for 48 h. The results showed that the silencing of TRAF3 in LUAD cells resulted in an evidently decrease in sensitivity to PTX (Fig. 4). The IC50 of PTX in A549 and H1299 cells treated with si-*TRAF*3 were 123.4 nM and 87.75 nM, while A549 and H1299 cells treated with si-NC were 43.39 nM and 38.39 nM, respectively. It is consistent with the previous conclusion that TRAF3 plays a tumor inhibitory role in lung adenocarcinoma.

### Downregulation of TRAF3 attenuates the pyroptosis of LUAD cells

**Fig. 5:**
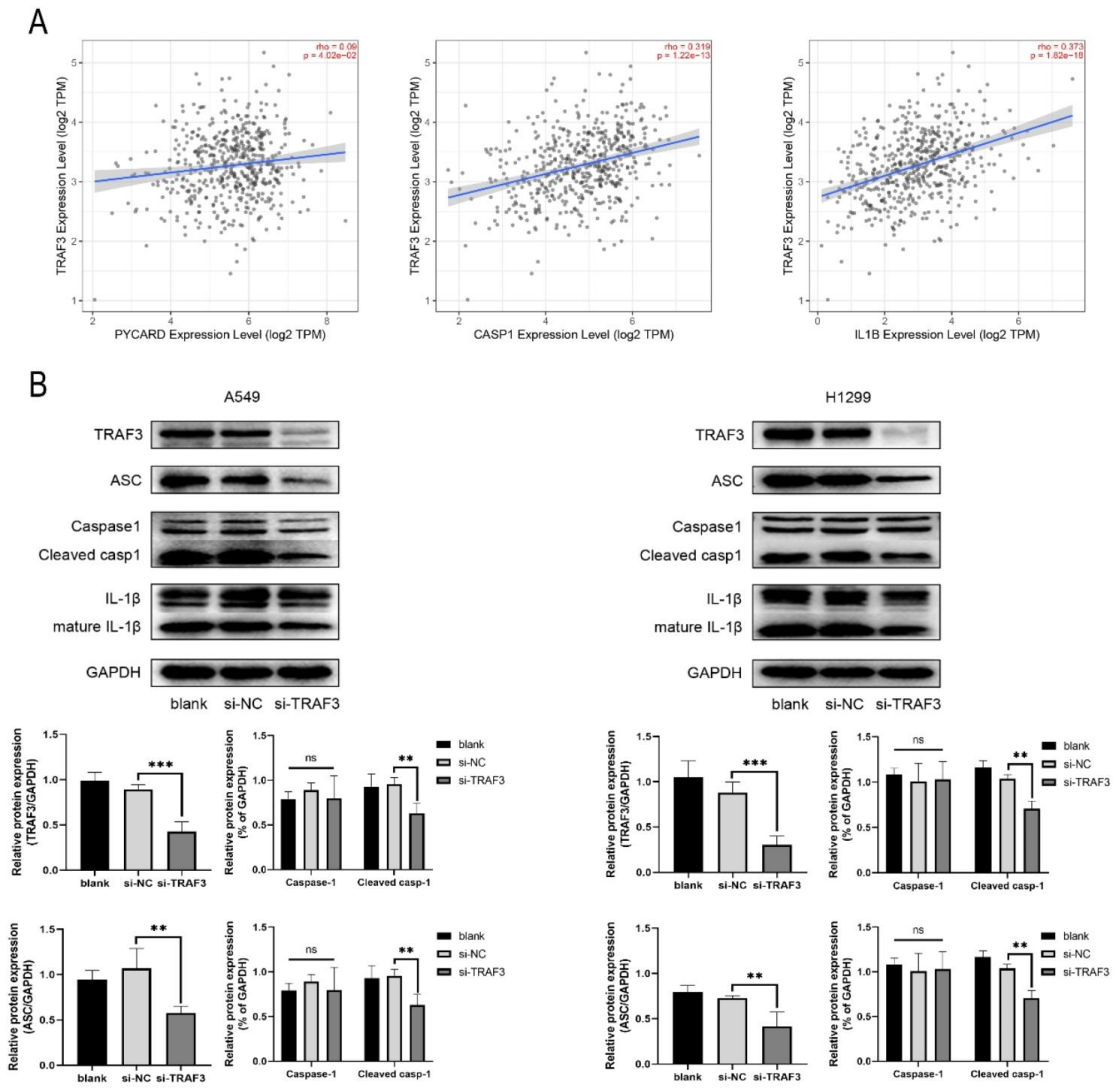
Downregulation of TRAF3 attenuates the pyroptosis of LUAD cells. **(A)** TIMER2.0 database predicted the association of TRAF3 with ASC, caspase-1 and IL-1β in lung adenocarcinoma. **(B)** After LUAD cells were transfected with TRAF3 siRNA, the expression levels of TRAF3, ASC, caspase-1, cleaved caspase-1, IL-1β and matured-IL-1β were analyzed by western blotting. *P<0.05, **P<0.01, ***P<0.001 versus control group. The results were presented as the mean ± SD (n = 3–4)

Pyroptosis, one type of programmed cell death, plays a vital role in maintaining the internal environment homeostasis and controlling the progression of many diseases. To verify the role of pyroptosis in lung adenocarcinoma and the involvement of TRAF3 in LUAD cells pyroptosis, we first queried the correlation between TRAF3 and pyroptosis-related genes. With the aid of TIMER2.0 database, we found that TRAF3 was positively correlated with the expression of ASC (R = 0.09, P<0.05), caspase-1 (R = 0.32, P<0.01) and IL-1β (R = 0.37, P<0.01) in lung adenocarcinoma (Fig. 5A). Subsequently, A549 and H1299 cells were treated with si-*TRAF*3 or si-NC to examine the expression level of pyroptosis-related protein. We observed a visibly decreased of the expression of ASC, cleaved caspase-1 and matured- IL-1β compared to those in the control group (Fig. 5B). Based on the existing results, we preliminarily concluded that TRAF3 plays a tumor inhibitory role in lung adenocarcinoma, in part by mediating caspase-1-dependent pyroptosis.

## Discussion

In the present study, we revealed that inhibition of TRAF3 promoted the proliferation and migration of lung adenocarcinoma cells. Inhibition of TRAF3 also weakened the sensitivity of lung adenocarcinoma cells to paclitaxel. The decrease in the expression of caspase-1-dependent pyroptosis related proteins suggests that TRAF3 may participate in the development of LUAD by mediating pyroptosis signals. As evidenced by these results, mediating TRAF3 is a promising treatment choice in lung adenocarcinoma.

Lung cancer, a group of diseases caused by abnormal cell proliferation and differentiation, is the leading cause of cancer death worldwide. Apoptosis resistance is a well-recognized hallmark of cancer, and numerous current treatments aim to induce apoptosis. In recent years, researchers have observed that some chemotherapeutic agents cause cytotoxicity through inducing pyroptosis or apoptosis-pyroptosis transition, providing new insights into cancer therapy [16]. Yu et al. [17] found that the colon cancer cells treated with lobaplatin were morphologically manifested as cell swelling and bubble bursting, and TEM showed the formation of cell membrane pores, which was consistent with the morphology of pyroptosis. Malignant mesothelioma (MM) exhibits decreased level of caspase-1, and doxorubicin induces inflammatory necrosis of MM cells by activating caspase-1 [18]. Pyroptosis induction also contributes to increased sensitivity to chemotherapy. Wu et al. [19] found that the Polo-like Kinase 1 (PLK1) kinase inhibitor BI2536 increased the sensitivity of oesophageal squamous cell carcinoma cells to cisplatin (DDP), combining with DDP could induce DNA damage and pyroptosis. It is consistent with our experimental results. When the level of pyroptosis is lowered, the proliferation and migration ability of LUAD cells is significantly enhanced, and the sensitivity of cells to paclitaxel is decreased.

TRAF3, one of the intracellular connective proteins, is widely expressed in eukaryotic cells, with specific modulation for a variety of signaling pathways. TRAF3-deficient mice die spontaneously at an early stage, suggesting that TRAF3 plays an important biological role in growth and development as well as in maintaining homeostasis of the internal environment [20]. Recently, more and more researches have been implied to clarify the relationship between TRAF3 and cancer. An increasing number of researches have shown that abnormal changes of TRAF3 gene were associated to abnormal cell growth and mutations and then leaded to tumor formation. Gema et al. [21] found a significant increase in the incidence of B cell hyperplasia and B cell lymphoma in B cell-specific TRAF3-deficient mice. In myeloid cell-specific TRAF3-deficient mice, myeloid-derived suppressor cells (MDSCs) were found to be significantly expanded, inhibiting the anti-tumor immune response mediated by T cells and promoting cell malignant transformation [14]. In human papillomavirus associated head and neck squamous cell carcinoma, TRAF3 not only inhibits NF-κB signal, simultaneously enhances the expression of TP53 and RB tumor suppressor proteins and promotes the apoptosis of cancer cells [22]. In addition, Shiode et al. [23] found that the TRAF3-NIK axis is related to the prognosis of human intrahepatic cholangiocarcinoma (ICC), and the inactivation of TRAF3 can promote the development of ICC through NIK-mediated hepatocyte transdifferentiation. The above studies indicate that TRAF3 plays a tumor inhibitory role in various types of cancer, providing a new breakthrough point for in-depth research on the pathogenesis and development of cancer. In recent years, investigators have discovered that the frequency of TRAF3 gene change in lung cancer is about 5.3%, which is closely related to the development and clinical prognosis of lung cancer [15]. However, the role of TRAF3 in non-small cell carcinoma remains unclear. Therefore, our group conducted some preliminary studies on the role of TRAF3 in lung adenocarcinoma. We found that TRAF3 was highly expressed in LUAD tissues and cells through bioinformatics and cellular assays. Nevertheless, Kaplan-Meier Plotter database showed that patients with TRAF3 hyperexpression in lung adenocarcinoma had significantly longer overall survival and progression-free survival compared to those with lower expression. In a series of subsequent functional experiments of CCK-8, Edu, cell scratching and transwell assays, inhibiting the expression of TRAF3 promoted the proliferation and migration of LUAD cells. As a result, we believe that TRAF3 may be a potential target for the treatment of LUAD.

Paclitaxel (PTX) is one of the most effective chemotherapy drugs in the clinical treatment of cancer. It inhibits the mitosis of cancer cells by affecting tubulin polymerization and microtubule stability, and finally induces cell apoptosis [24]. PTX has been ratified by Food and Drug Administration for the treatment of diverse cancers, including lung cancer, ovarian cancer, breast cancer and lymphoma [25]. Although PTX has shown promising therapeutic effect in lung cancer, it is limited by the drug resistance, mainly due to the overexpression of P-glycoprotein or altered tubulin [26]. Seeking biomarkers of lung cancer and improving the efficacy of chemotherapy is of great significance. Therefore, we further investigated the association between PTX and TRAF3. We observed that silencing TRAF3 significantly decreased the sensitivity of lung adenocarcinoma cells to PTX, indicating that TRAF3 and PTX have synergistic antitumor effect.

Previous studies have shown that TRAF3 is a critical regulator of pyroptosis, and TRAF3 is involved in the development of various diseases by regulating the pyroptosis pathway. Therefore, we speculate that TRAF3 may exert tumor suppressive effects through the regulation of pyroptosis signaling. We first analyzed the correlation between TRAF3 and pyroptosis-related genes through TIMER2.0 database, the results showed that TRAF3 was positively correlated with the expression of ASC, caspase-1 and IL-1β in lung adenocarcinoma. In addition, cellular assays also showed that inhibition of TRAF3 significantly downregulated the expression levels of ASC, cleaved caspase-1 and matured-IL-1β. It suggests that TRAF3 may inhibit the development of lung adenocarcinoma by regulating the caspase-1-dependent pyroptosis signaling. Our findings are consistent with previous studies. Shen et al. [27] showed that TRAF3 plays a pivotal role in the mitochondrial reactive oxygen species production, inflammasomes activation and cell inflammatory death. Shen at al [28] revealed that TRAF3 is a new E3 ubiquitin ligase of ASC, which stabilizes and elevates ASC levels by inducing K63-linked ubiquitination, thereby promoting inflammasome activation. In conclusion, TRAF3 maybe function as tumor suppressor by modulating the caspase-1-dependent pyroptosis signaling pathway.

Currently, our experiments have only unilaterally investigated the effect of TRAF3 on the development of LUAD cells from downregulation of TRAF3. The effect of overexpression of TRAF3 on lung adenocarcinoma needs to be clarified. Moreover, the specific targets of TRAF3 action on the pyroptotic pathway need to confirm by further in vivo and in vitro experiments.

## Conclusions

In conclusion, inhibiting of TRAF3 not only promoted the proliferation and migration of lung adenocarcinoma, but weakened the reactive sensitivity of the cells to paclitaxel. Inhibition of TRAF3 affected the expression levels of ASC, cleaved caspase-1 and matured-IL-1β, supporting a regulatory role of TRAF3 in pyroptosis signaling pathway. This study provides new insights into the effects and regulating mechanism of TRAF3 on lung adenocarcinoma, which may carry important clinical implications for the personalized treatment of lung cancer.

### Electronic supplementary material

Below is the link to the electronic supplementary material.

Supplementary Material 1

## Data Availability

The datasets used or analysed during the current study are available from the corresponding author on reasonable request.
