# Supplementary material for: Effects of TRAF3 on the proliferation and migration of lung adenocarcinoma depend partly on pyroptosis
Source: BMC Cancer. 2023 Oct 5;23:942. doi: 10.1186/s12885-023-11468-z (PMC10557279; doi:10.1186/s12885-023-11468-z)
Supplement: Supplementary file 1 — Supplementary Material 1 [file 12885_2023_11468_MOESM1_ESM.docx]

**Fig.1**

Repeat1 Repeat2 Repeat3


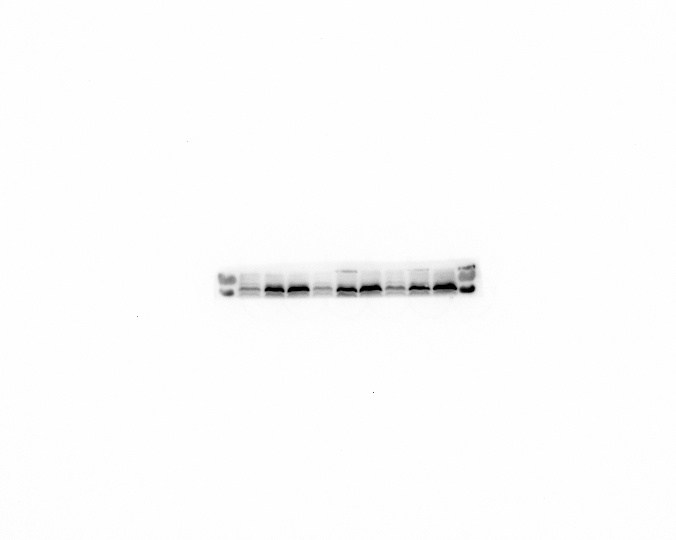


55

70

TRAF3 64kDa


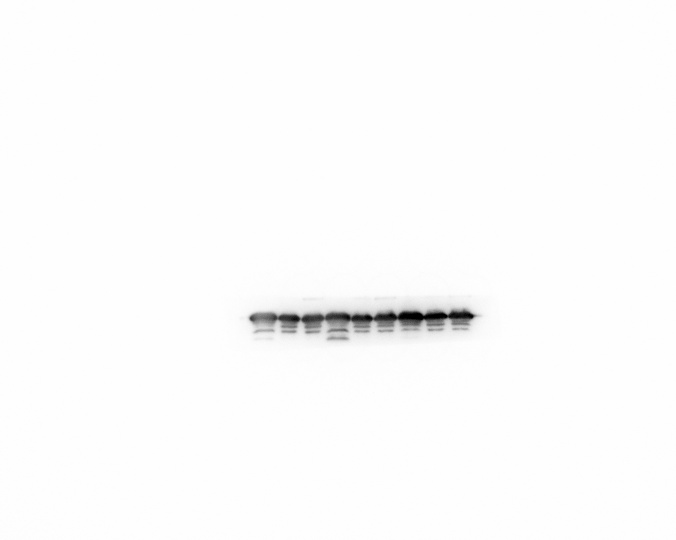


GAPDH 37kDa

16HBE

16HBE

16HBE

A549

A549

H1299

H1299


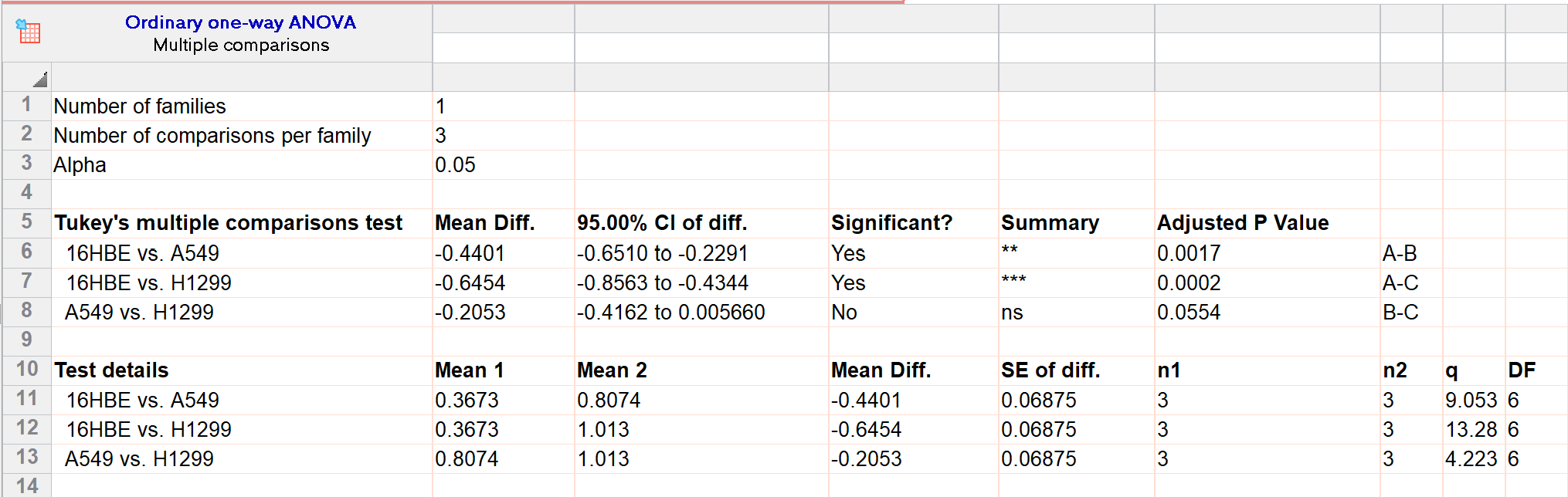

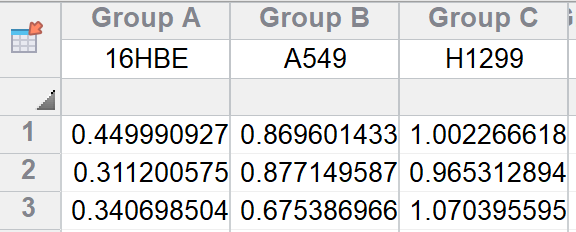


35

H1299

A549

**
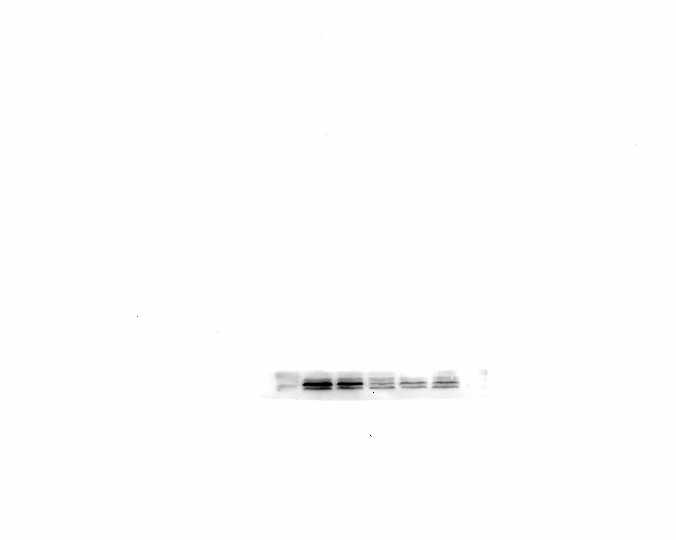
Fig.2**

Repeat1

55

70

TRAF3 64kDa

GAPDH 37kDa

**
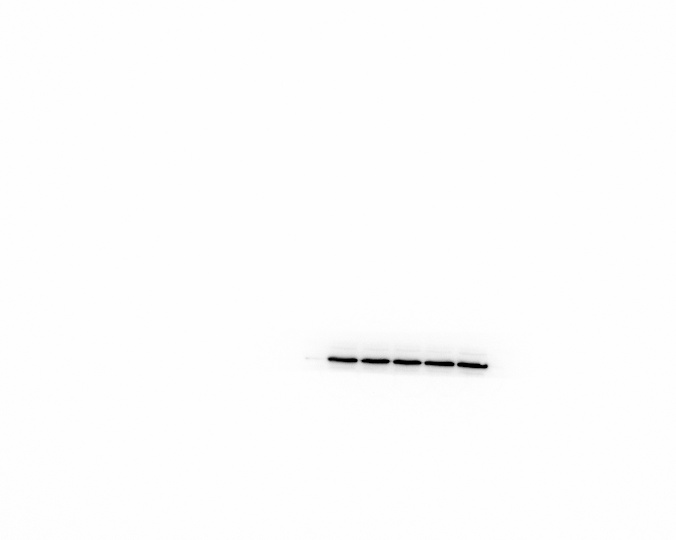
**

35


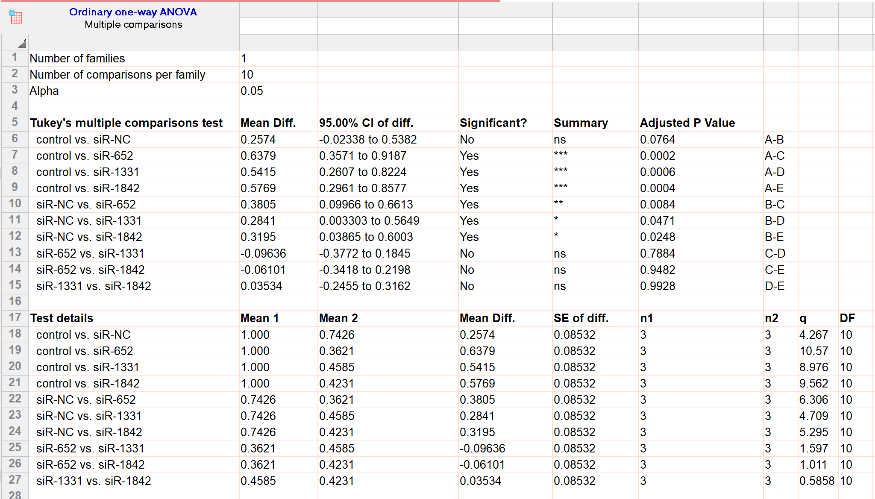
**
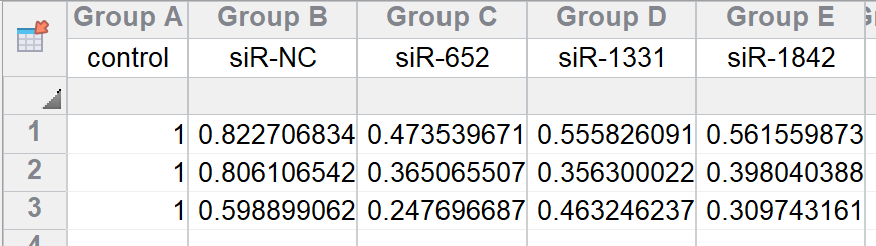
**
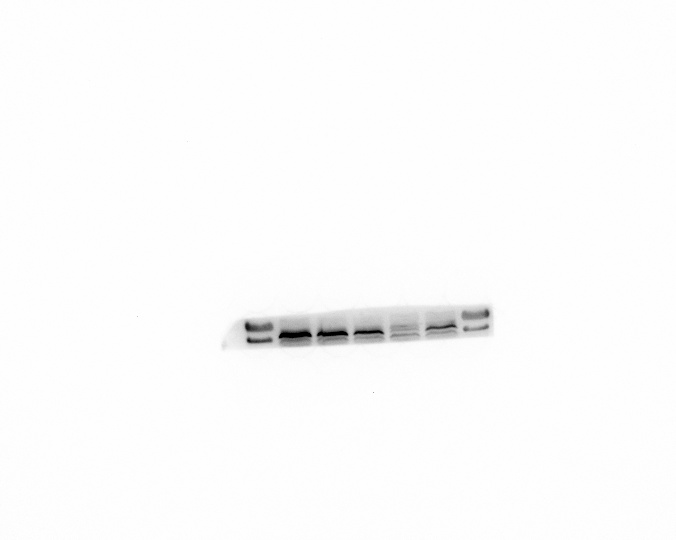

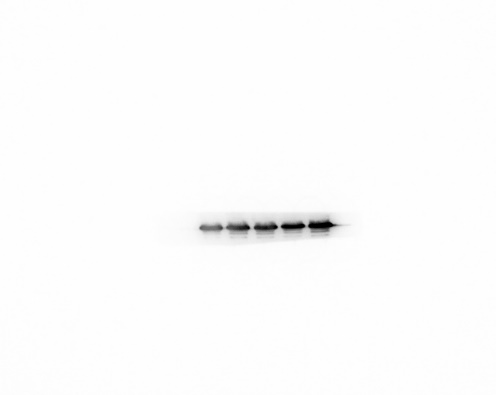

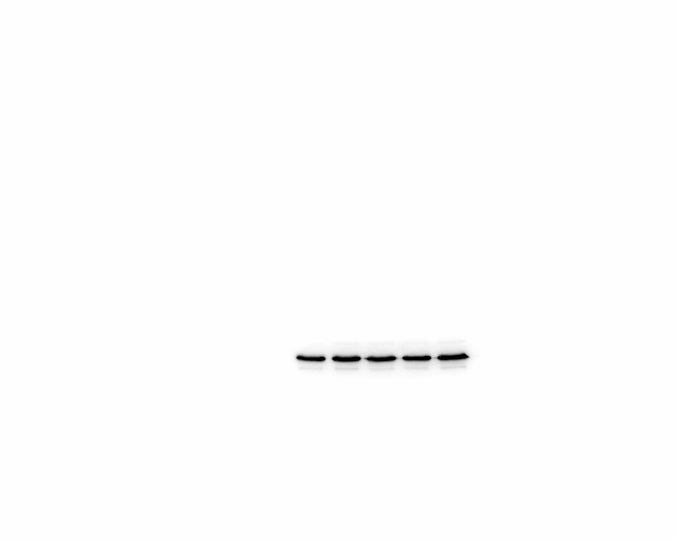

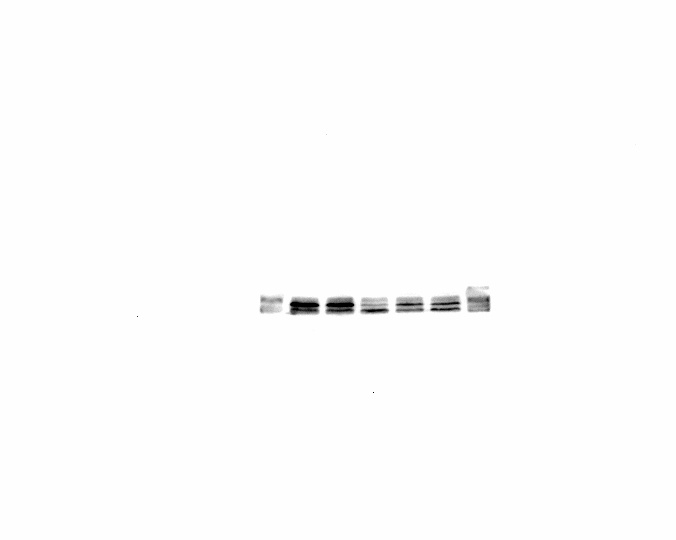


control siR-NC siR-1331 siR-652 siR-1842

Repeat3

TRAF3 64kDa

70

55

GAPDH 37kDa

35

control siR-NC siR-652 siR-1331 siR-1842

Repeat2

TRAF3 64kDa

70

55

GAPDH 37kDa

35

control siR-NC siR-652 siR-1331 siR-1842

**Fig.5**


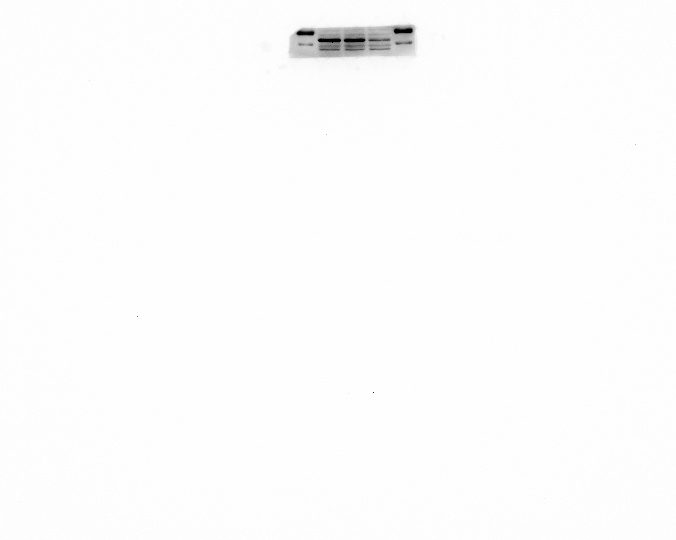
**
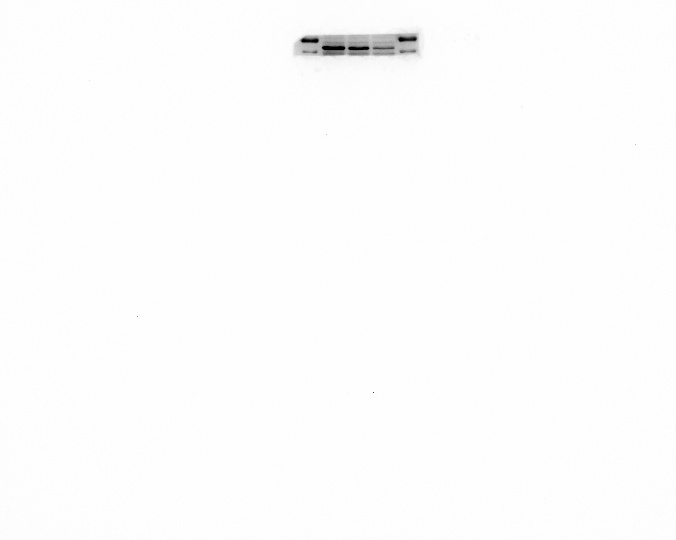
A549**

Repeat2

Repeat1

TRAF3 64kDa

70

55

**
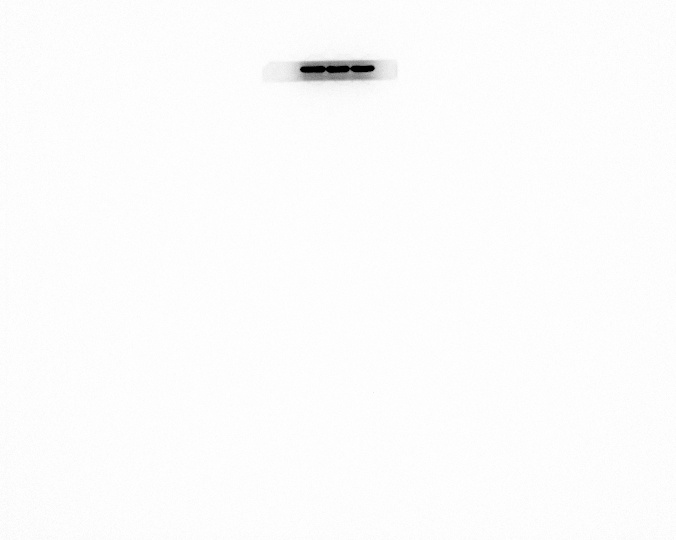
**
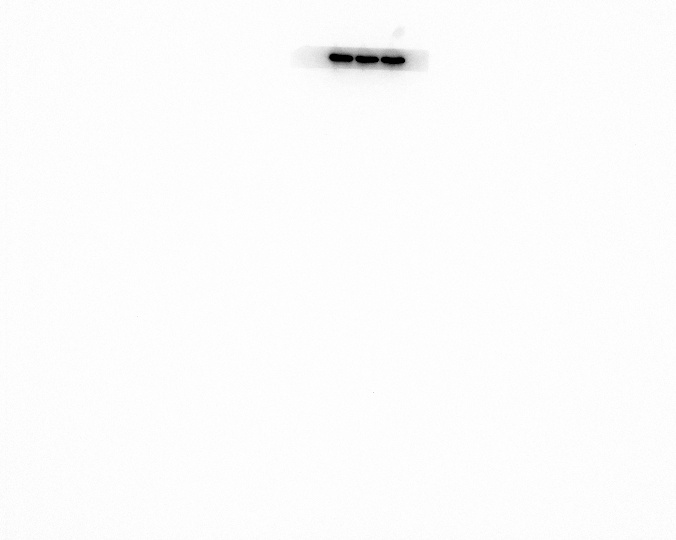


GAPDH 37kDa

control si-NC si-TRAF3

35

**
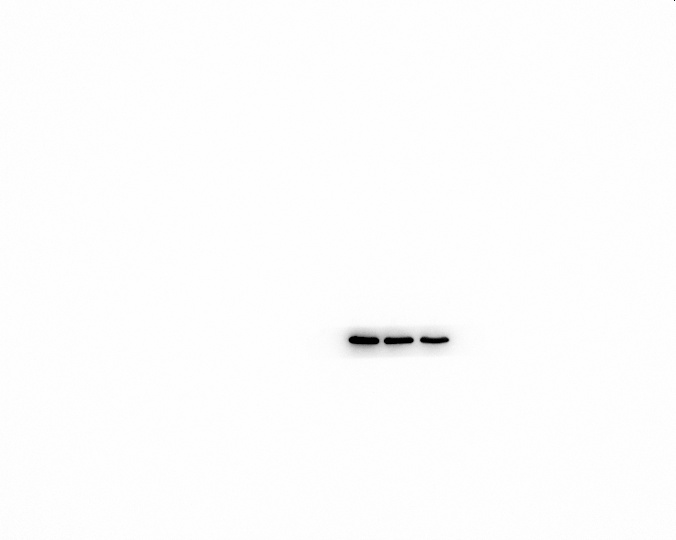

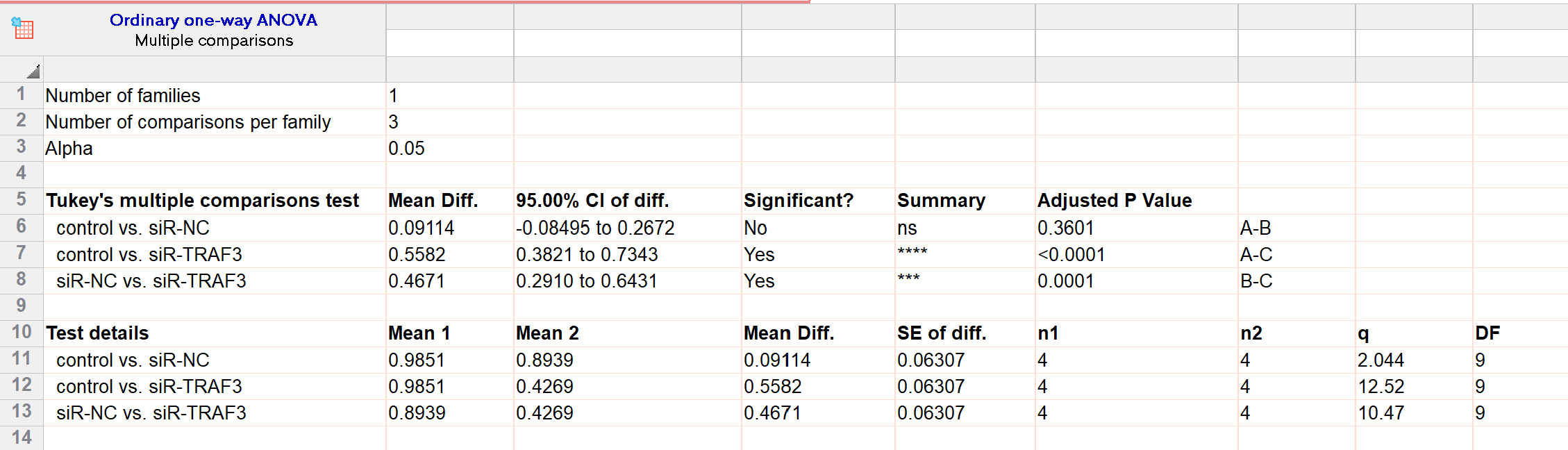

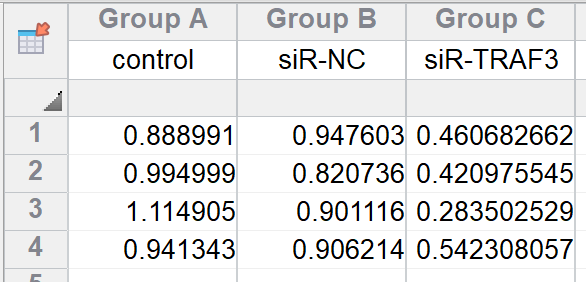

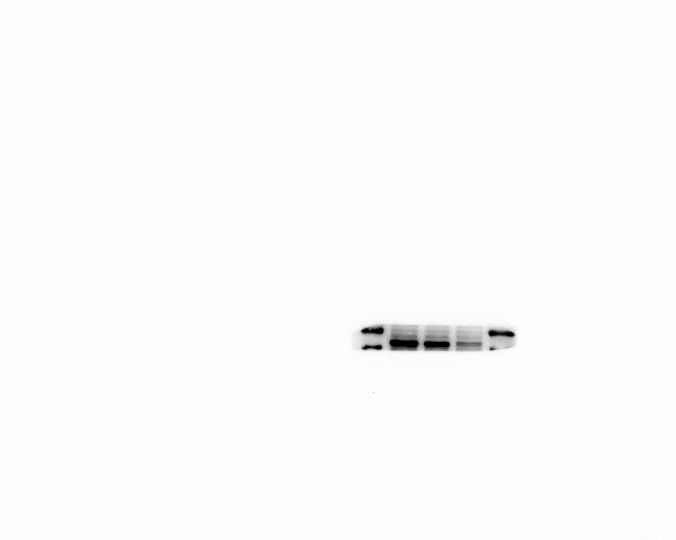

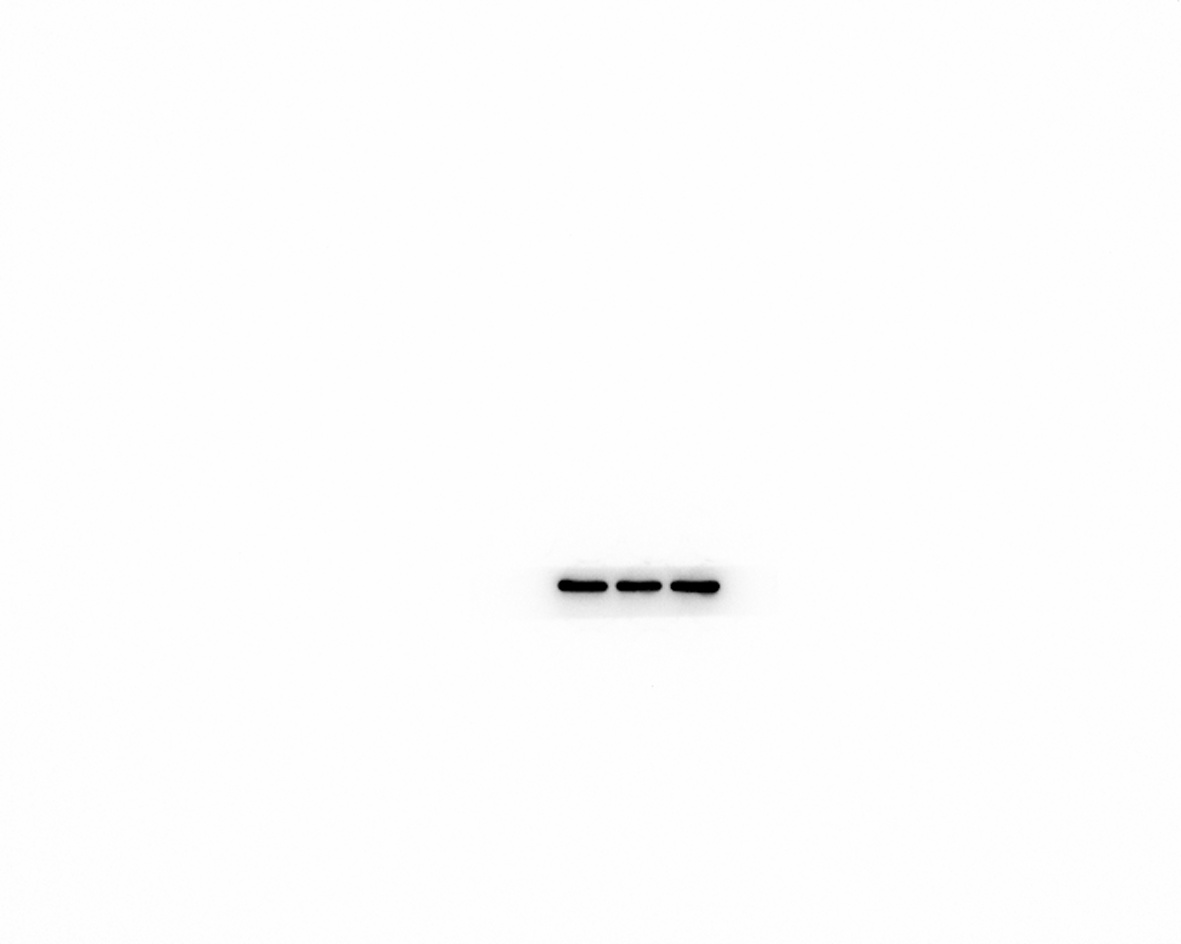

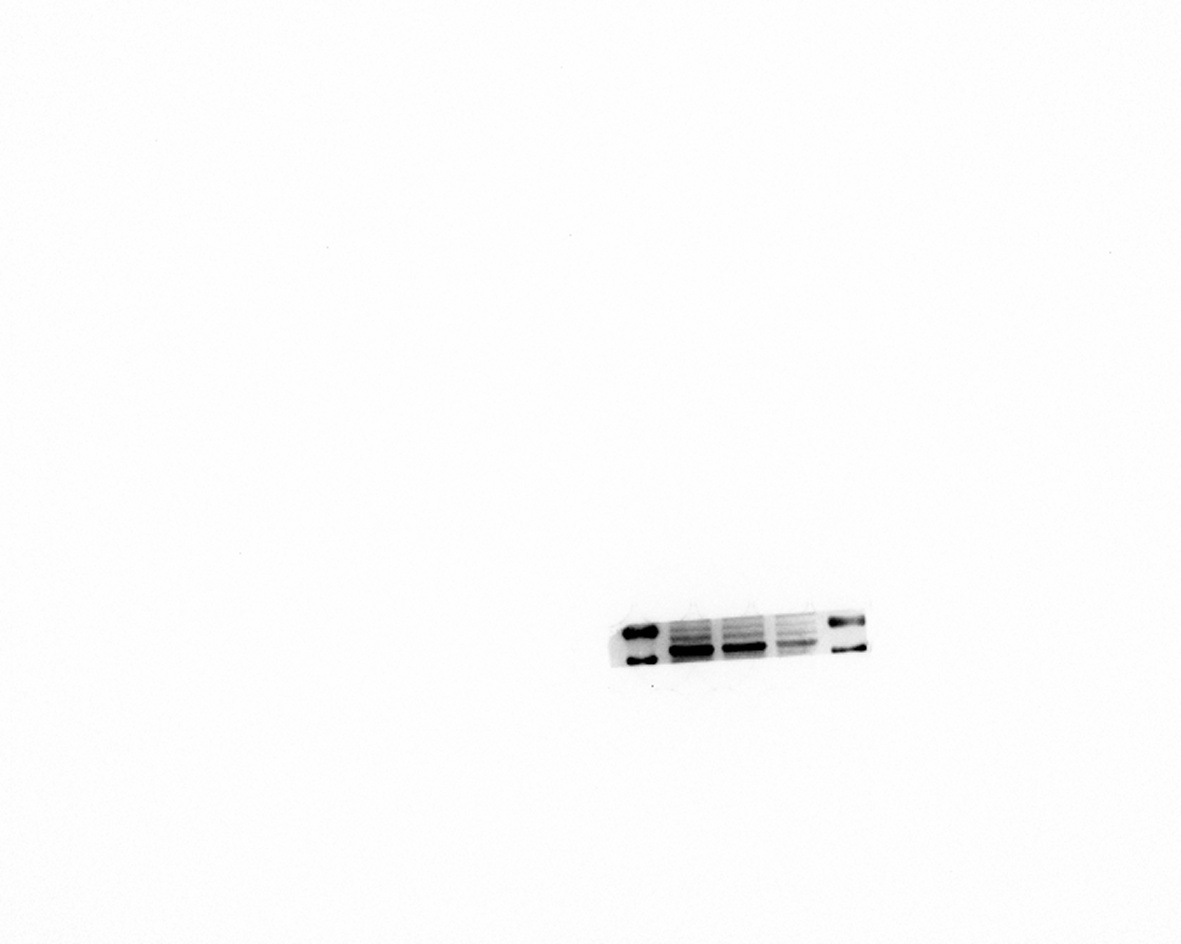
**

control si-NC si-TRAF3

control si-NC si-TRAF3

Repeat4

Repeat3

control si-NC si-TRAF3

35

GAPDH 37kDa

55

70

TRAF3 64kDa

**Fig.5**

**
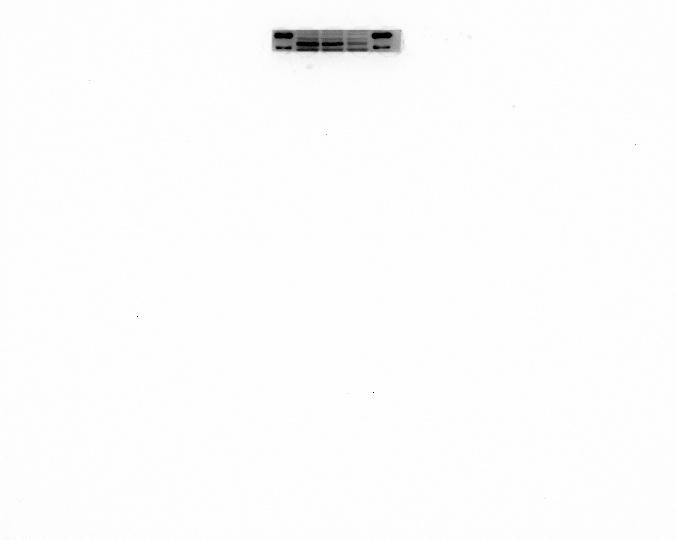

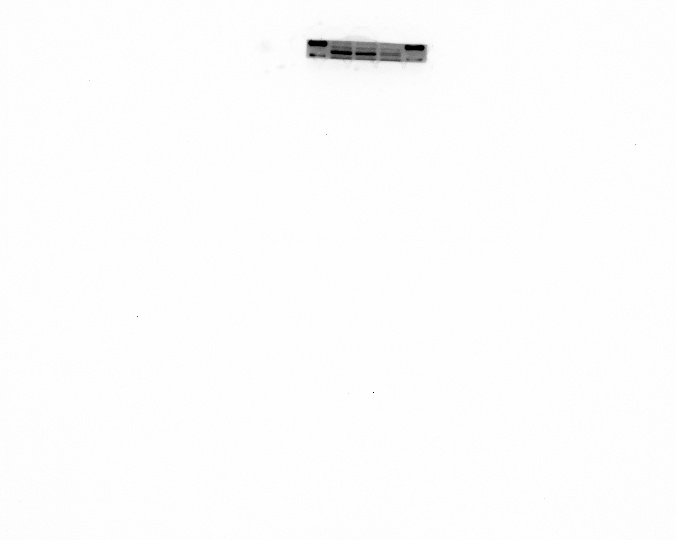
H1299**

Repeat1

Repeat2

TRAF3 64kDa

70

55

GAPDH 37kDa

35

TRAF3 64kDa

70

55

GAPDH 37kDa

35

control si-NC si-TRAF3

**
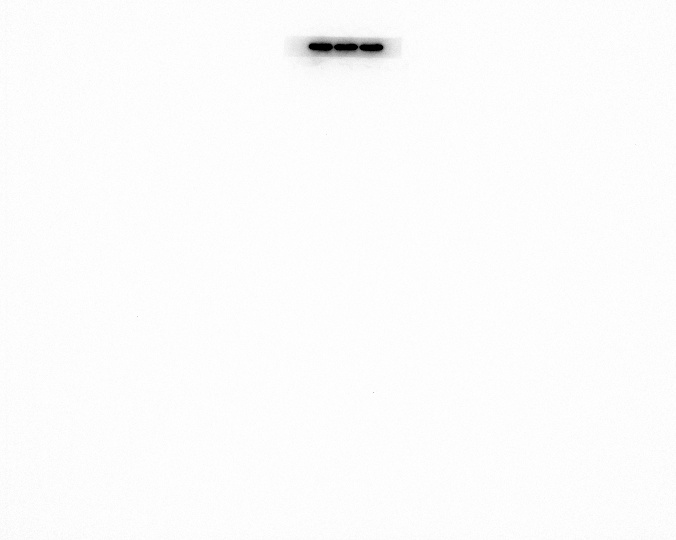
**

**
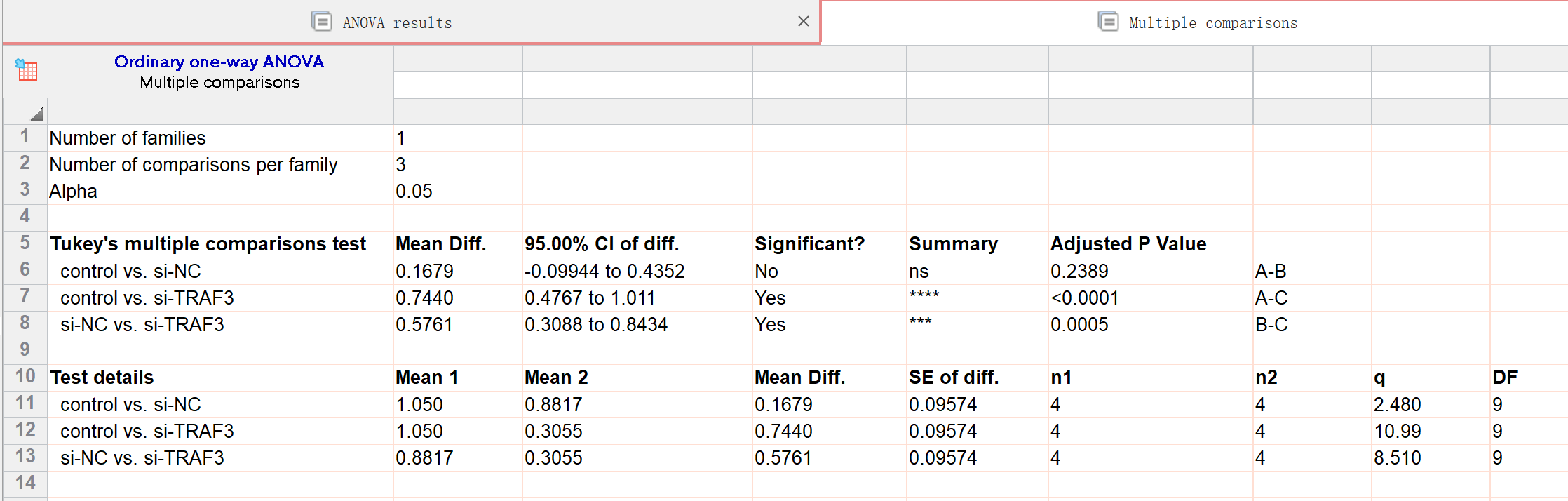

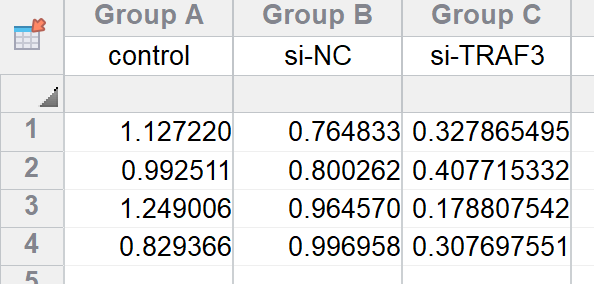

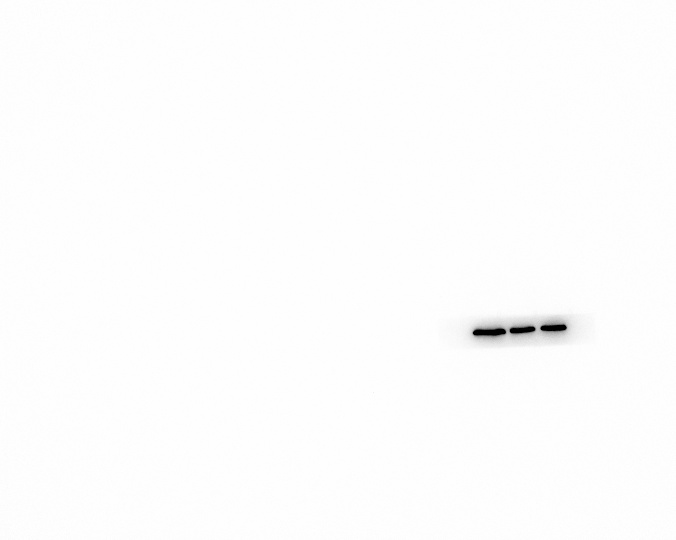

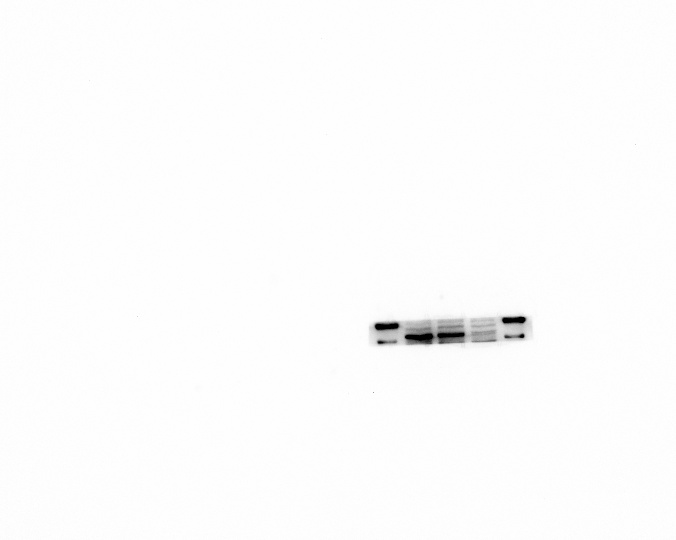

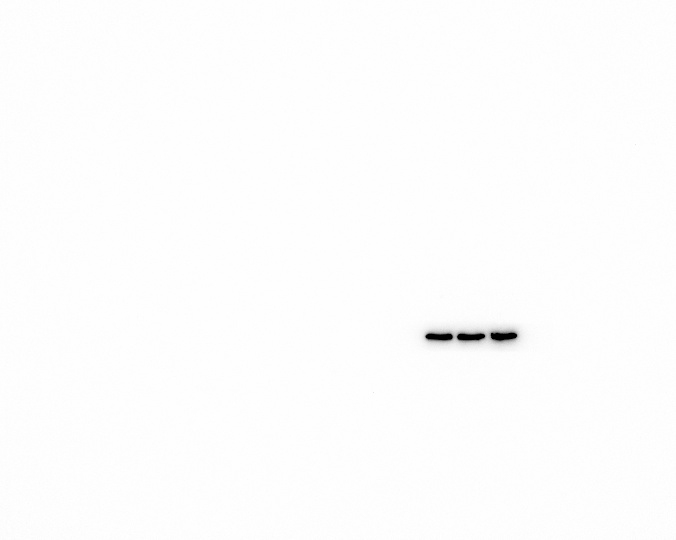

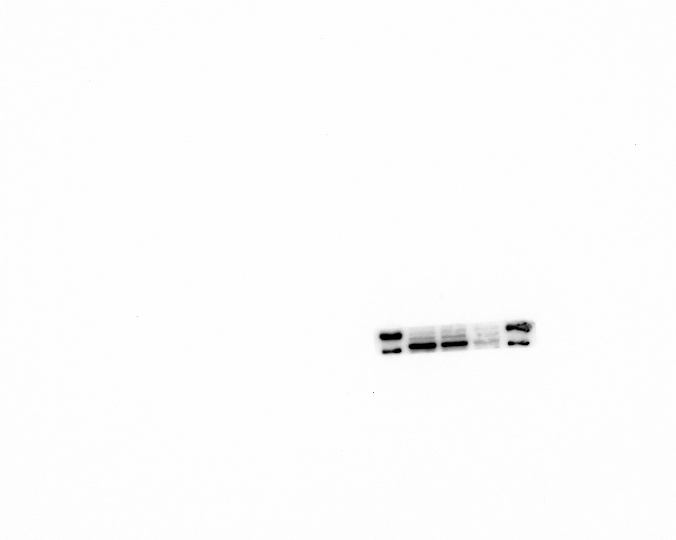

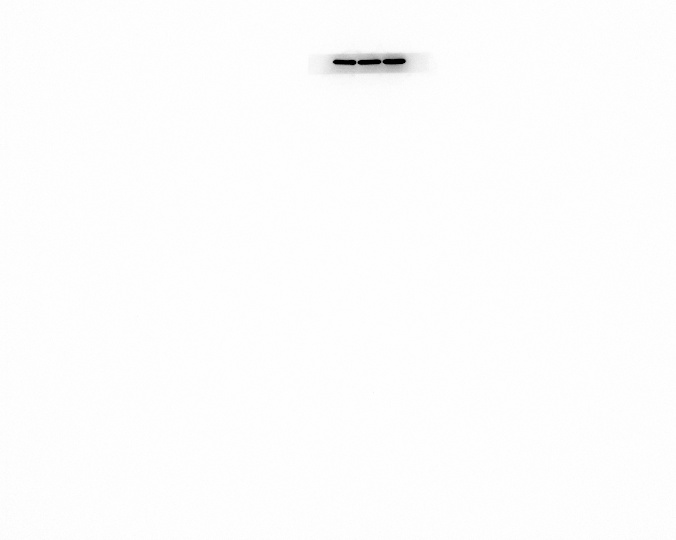
**

Repeat4

control si-NC si-TRAF3

Repeat3

control si-NC si-TRAF3

control si-NC si-TRAF3

**Fig.5**

**
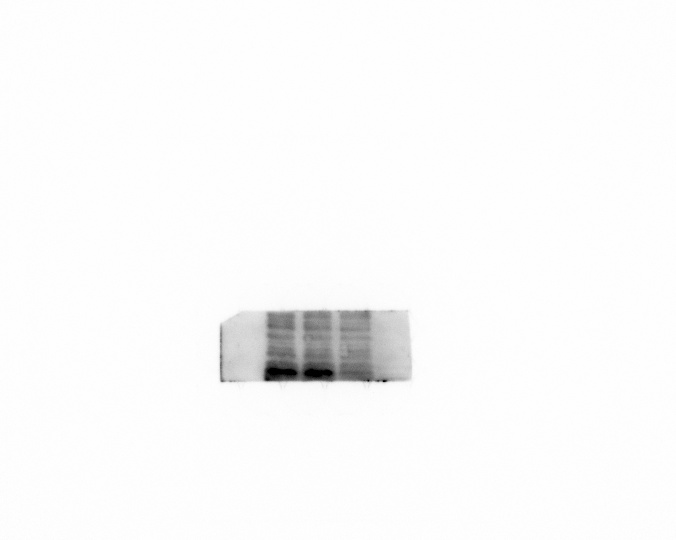

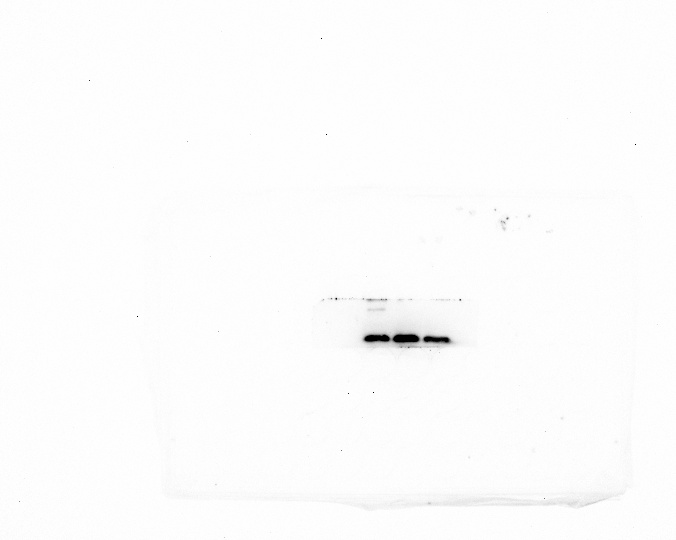
A549**

Repeat2

25

Repeat1

**
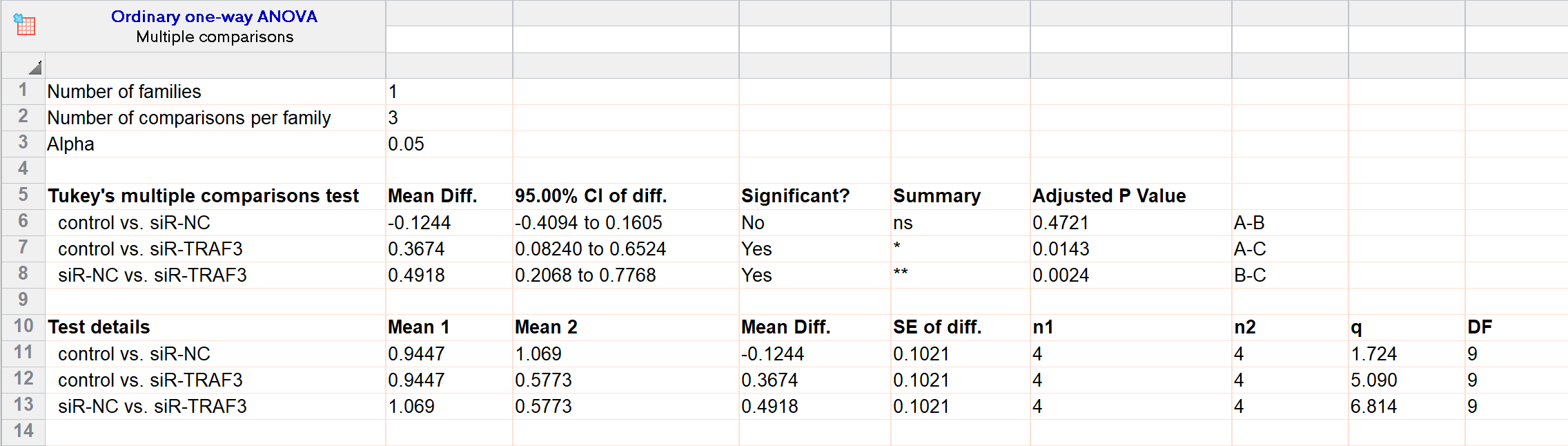

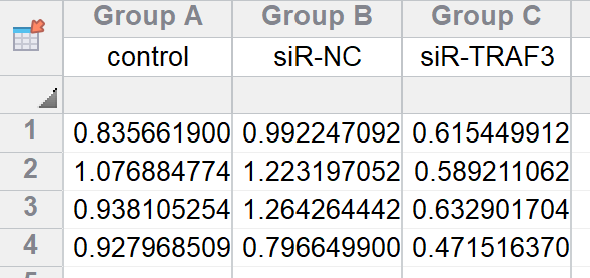

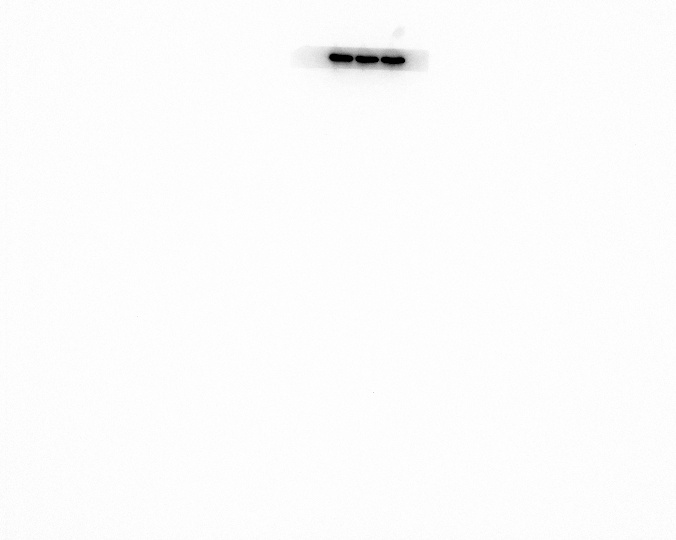

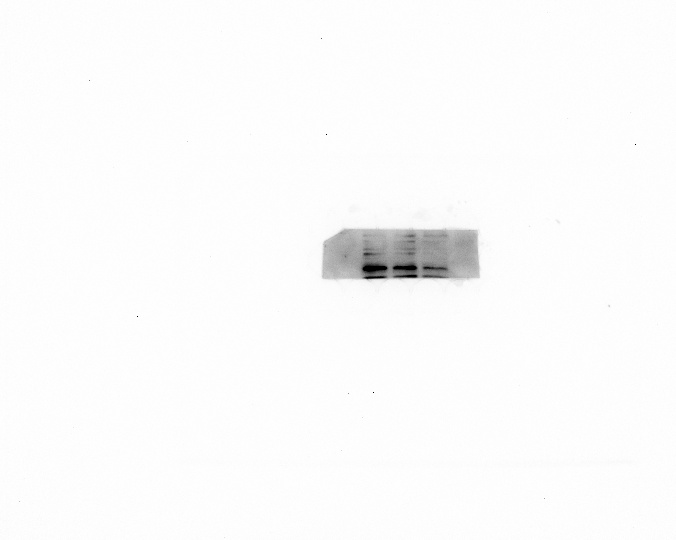

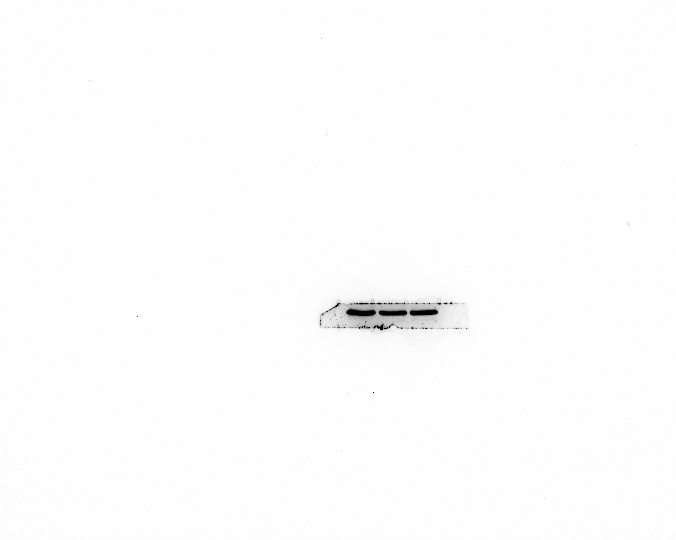

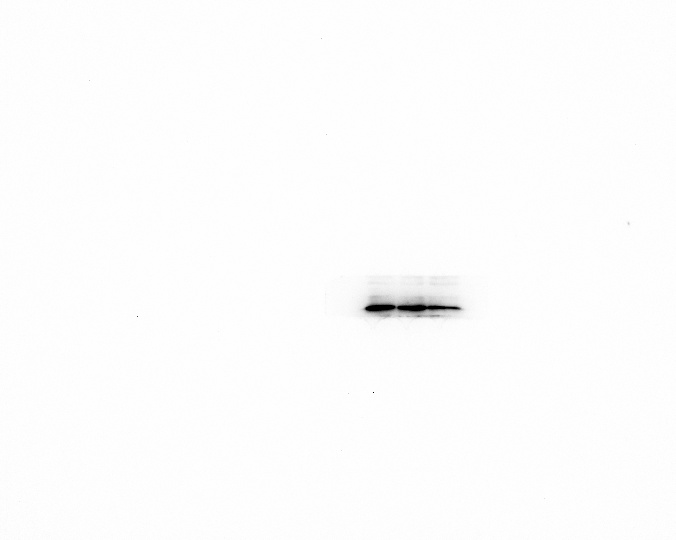

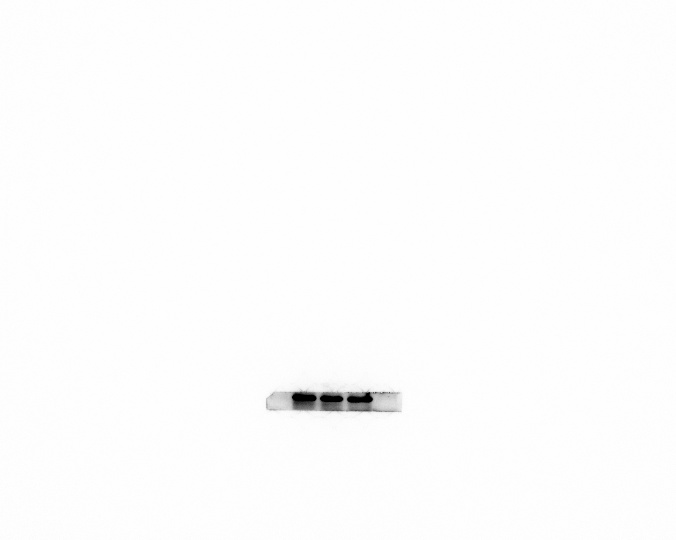

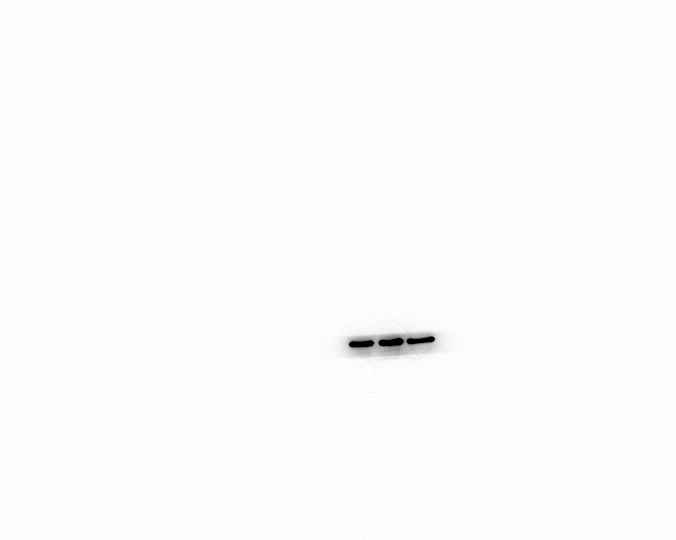
**

control si-NC si-TRAF3

control si-NC si-TRAF3

control si-NC si-TRAF3

control si-NC si-TRAF3

Repeat3

ASC 19kDa

25

Repeat4

GAPDH 37kDa

35

35

GAPDH 37kDa

ASC 19kDa

**Fig.5**

**H1299**

Repeat1

ASC 19kDa

25

Repeat2

GAPDH 37kDa

35

Repeat3

ASC 19kDa

25

Repeat4

GAPDH 37kDa

35

**
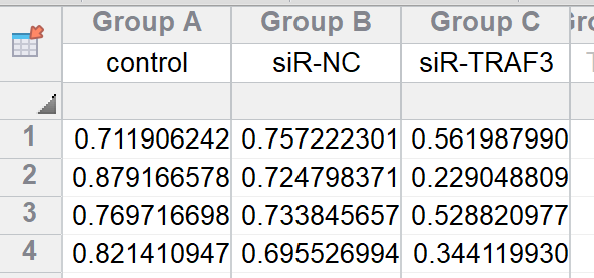

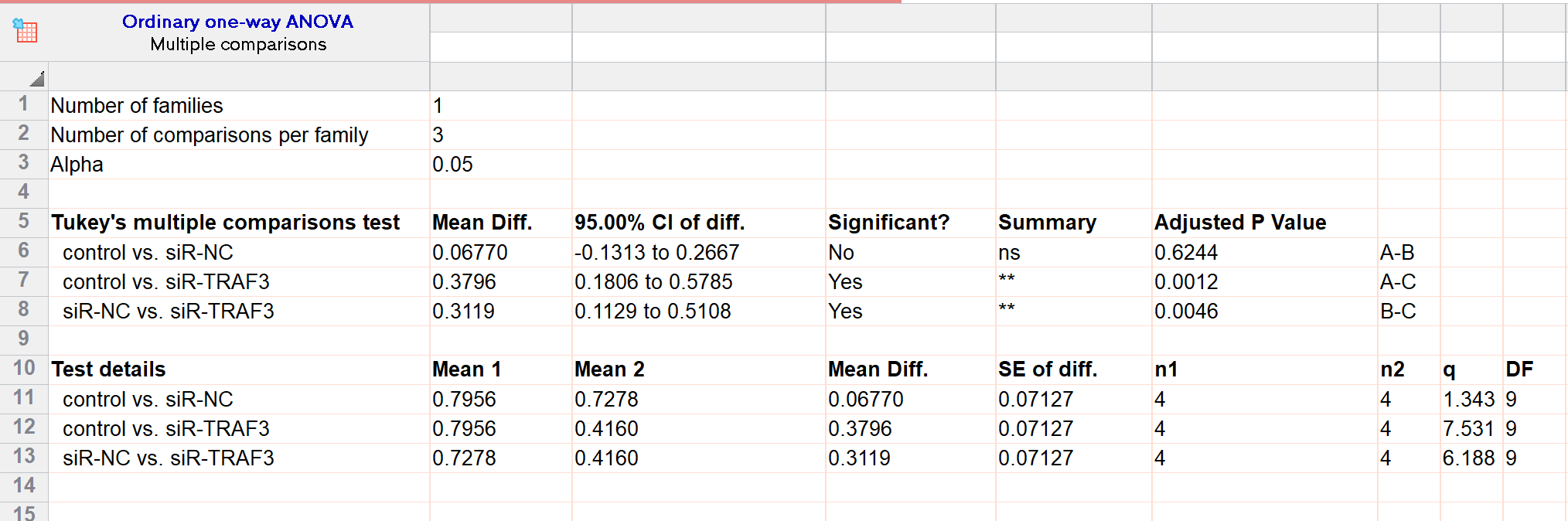

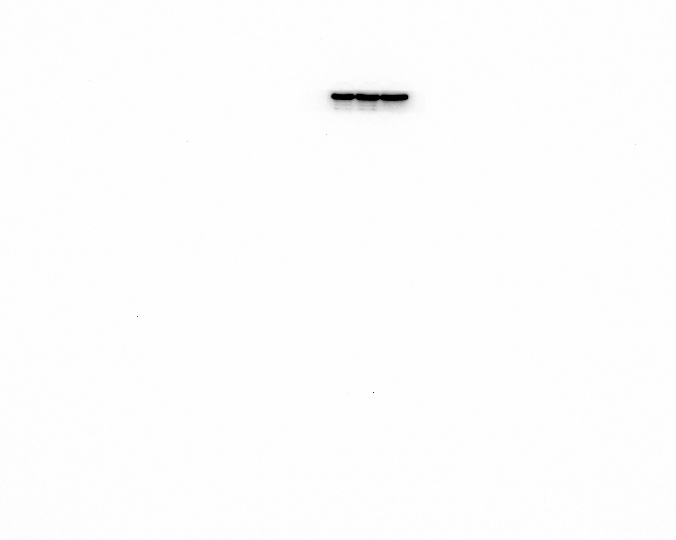

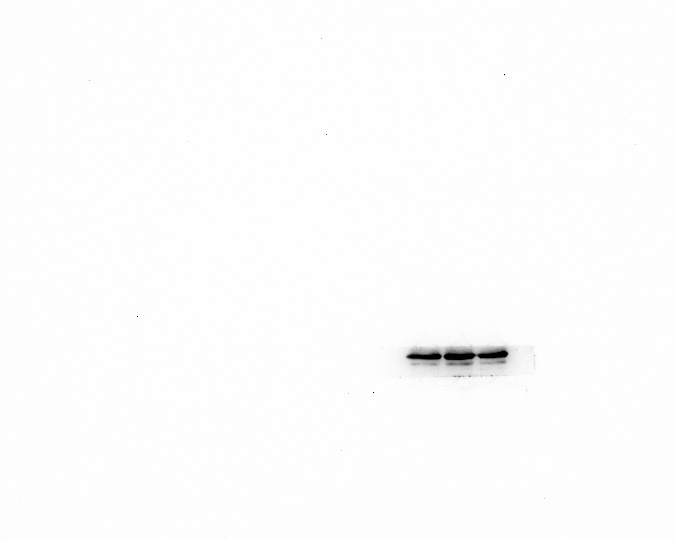

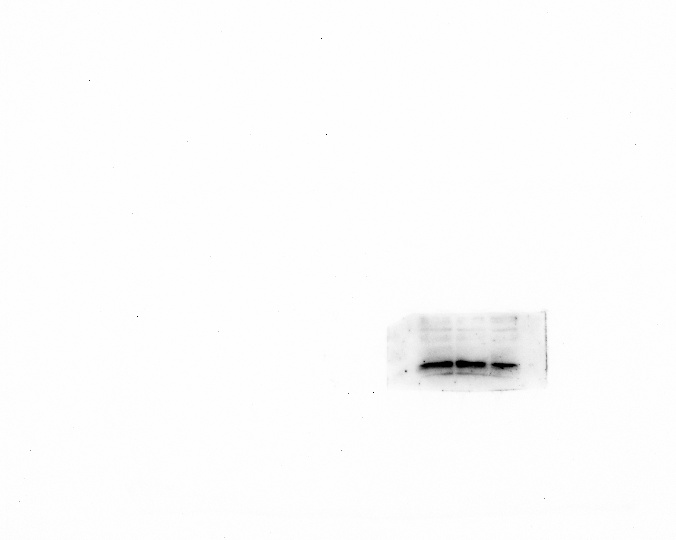

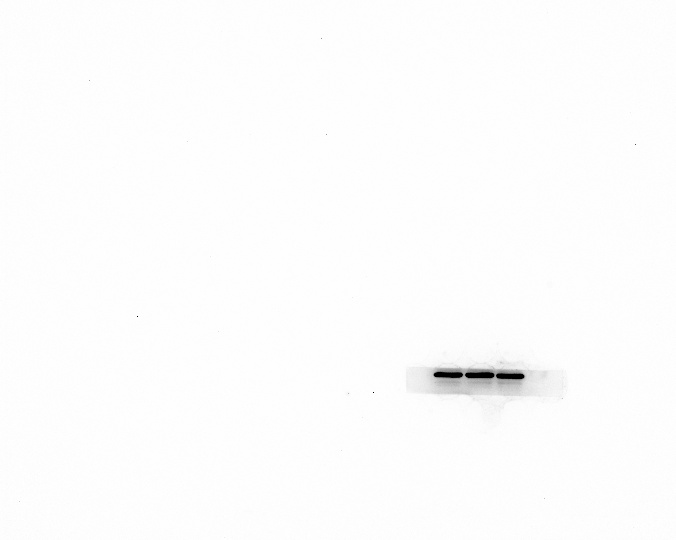

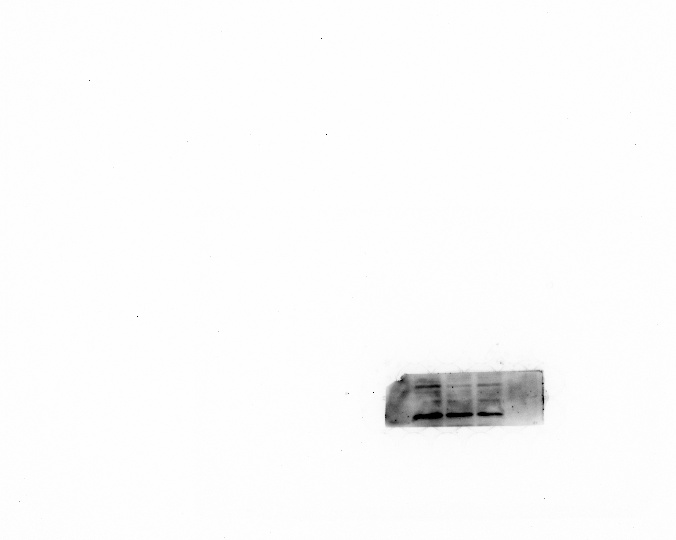

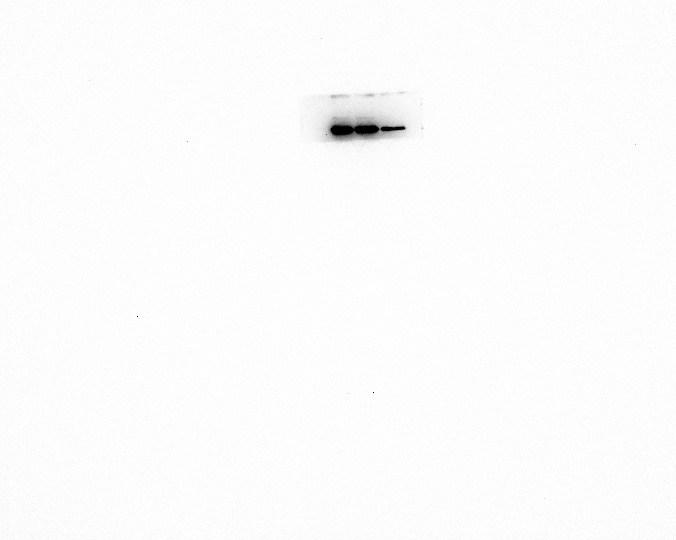

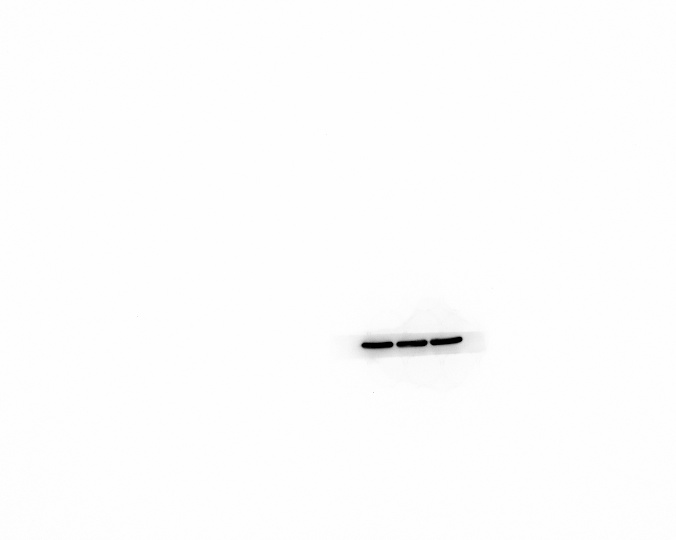
**
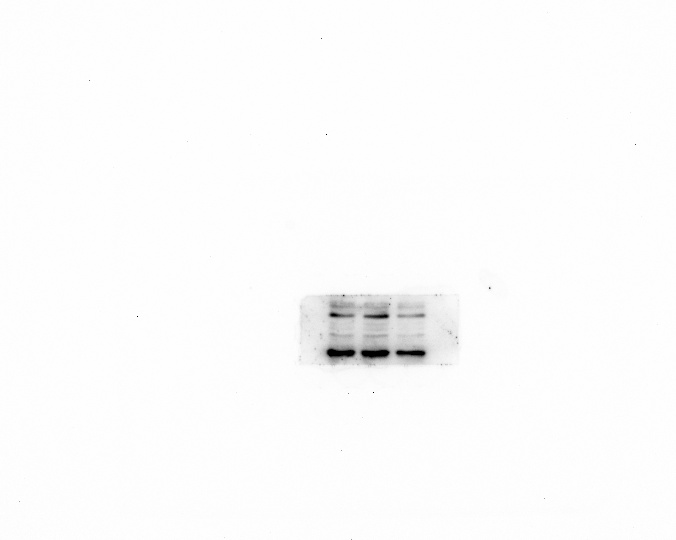


35

control si-NC si-TRAF3

control si-NC si-TRAF3

control si-NC si-TRAF3

control si-NC si-TRAF3

**Fig.5**

**
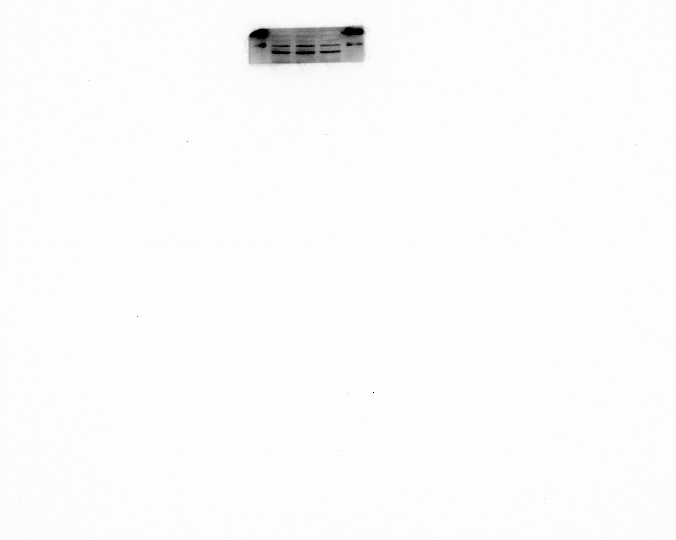

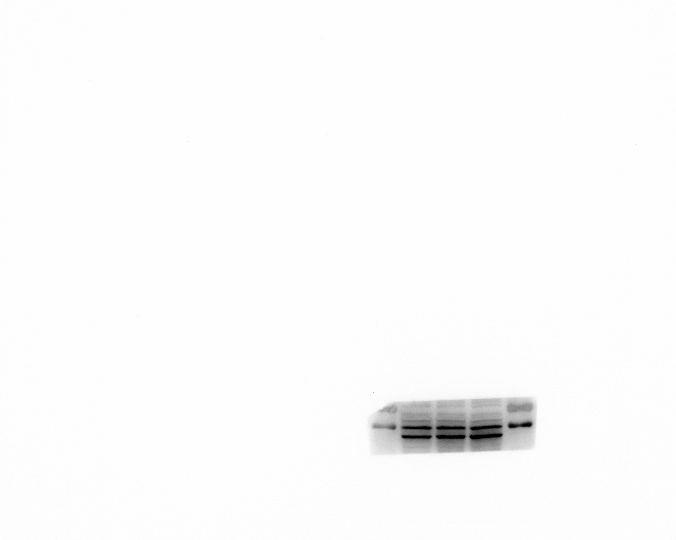
A549**

Repeat1

Repeat2

Caspase1 45、42kDa

55

**
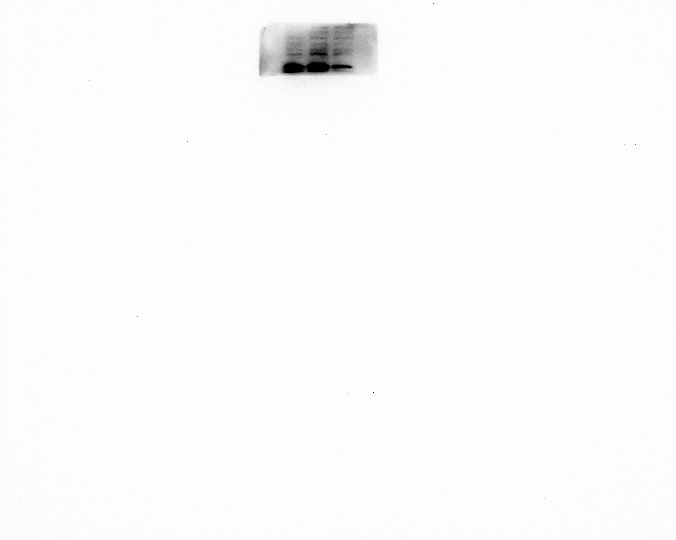

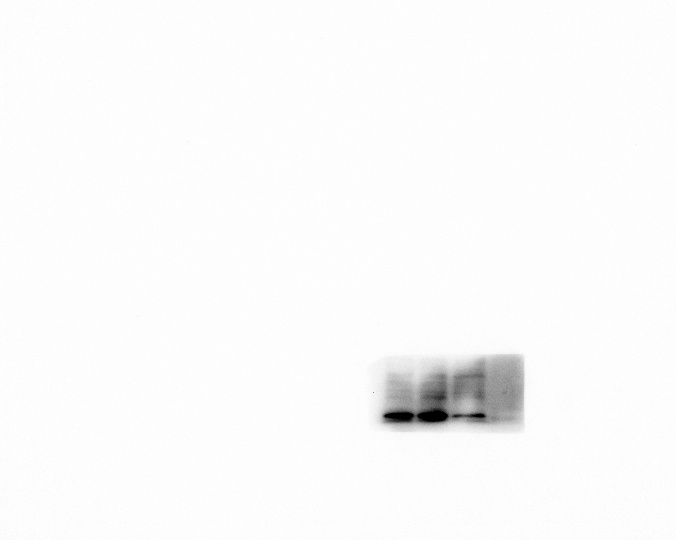
**

Cleaved casp1 12、10kDa

25

**
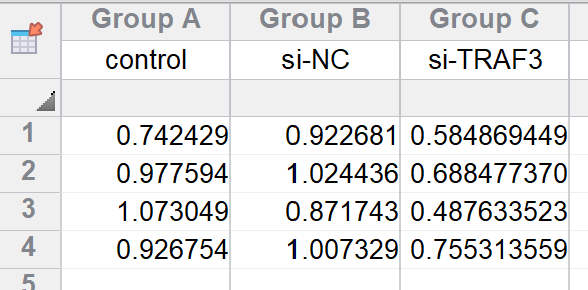

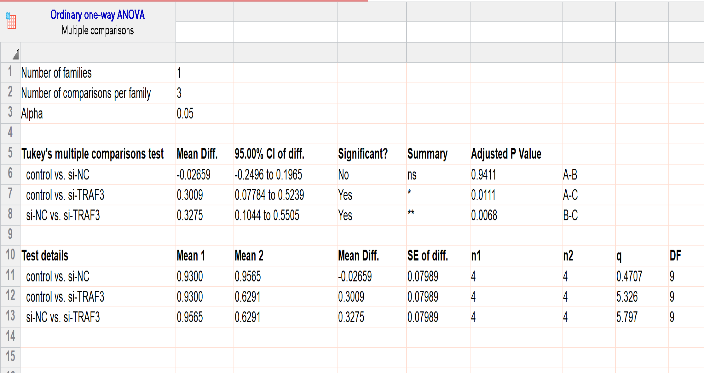

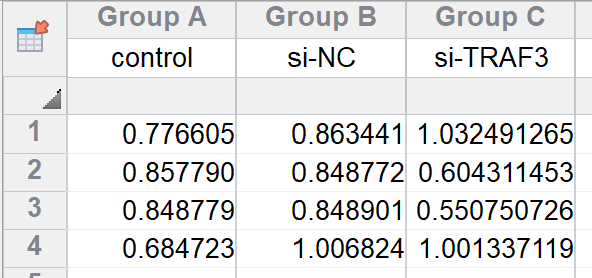

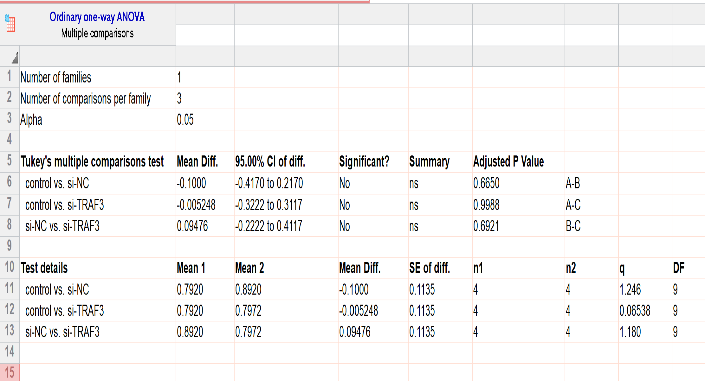
**
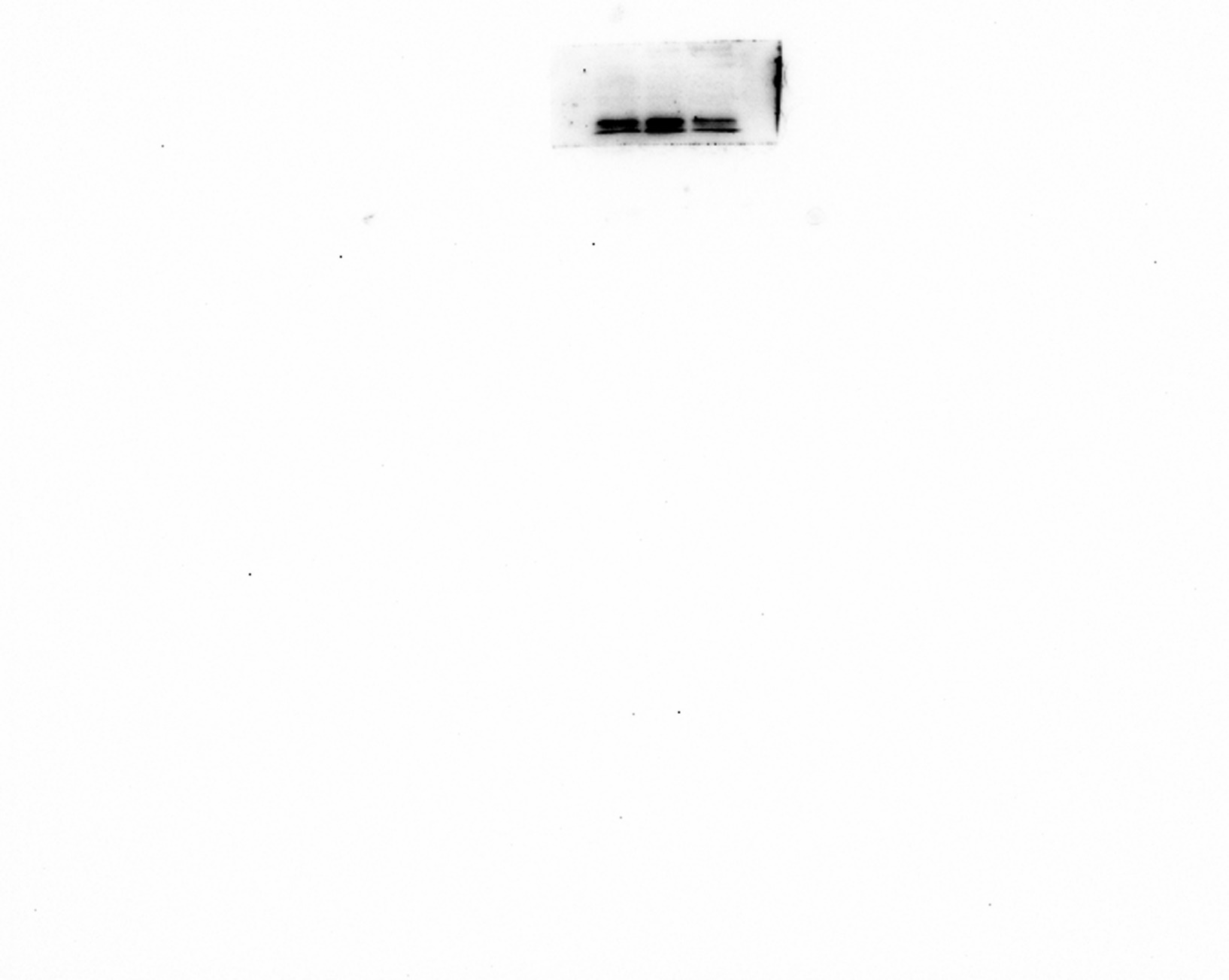
**
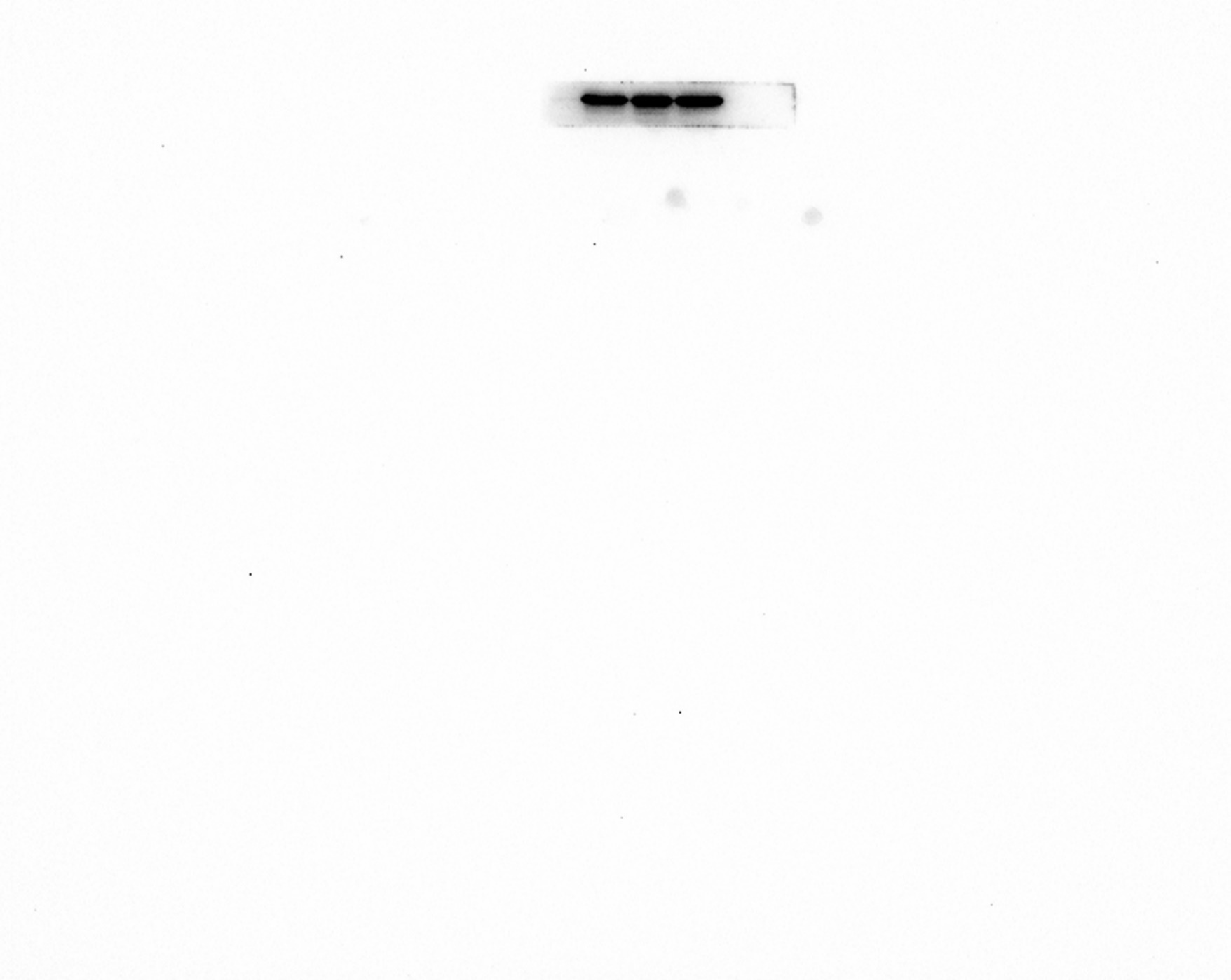

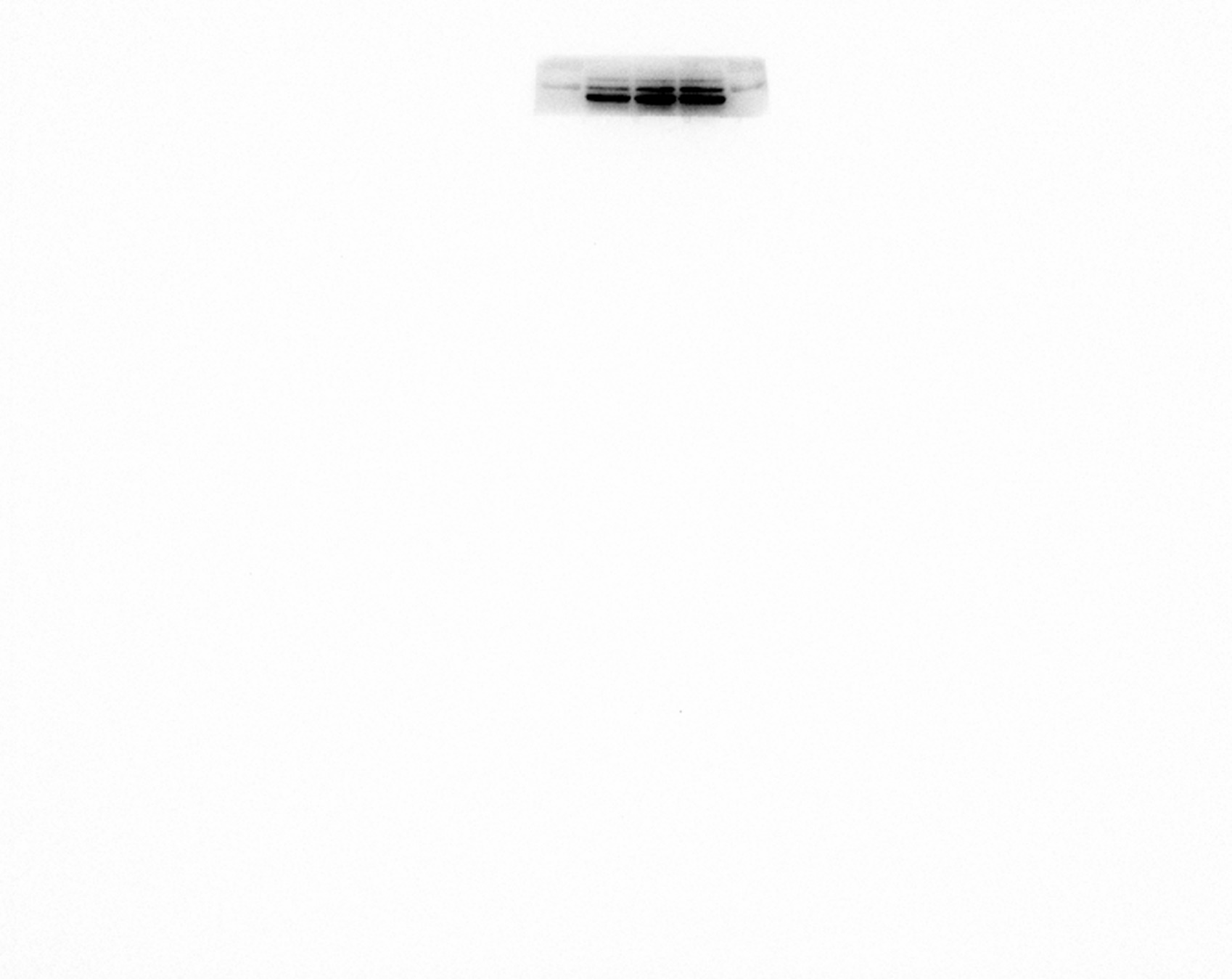

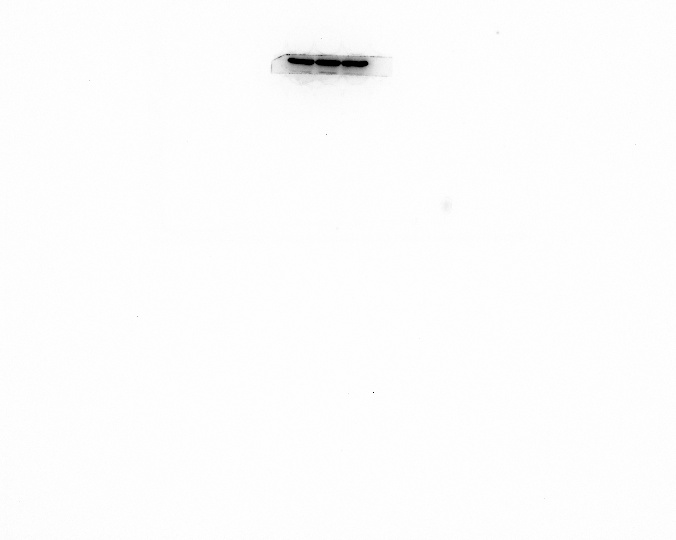

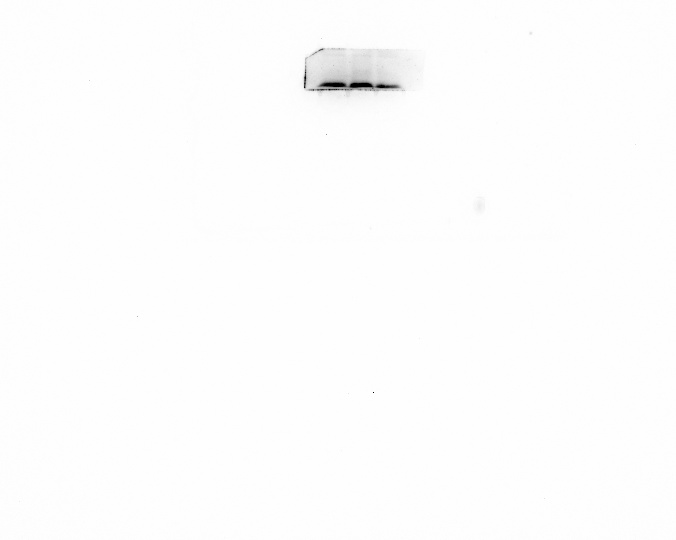

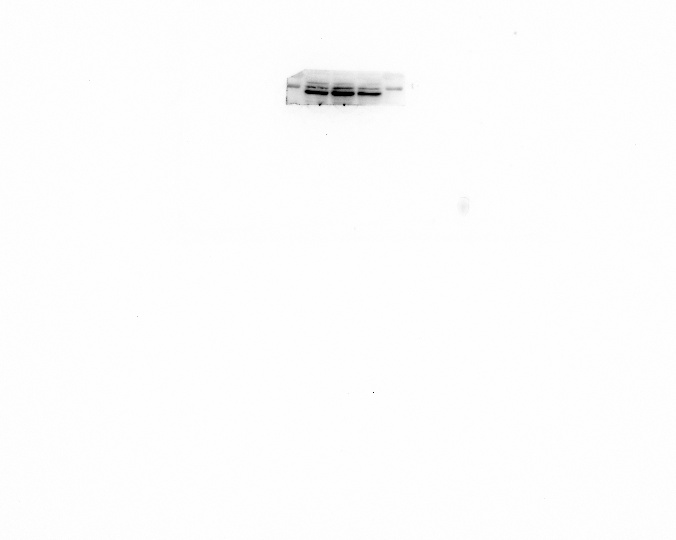

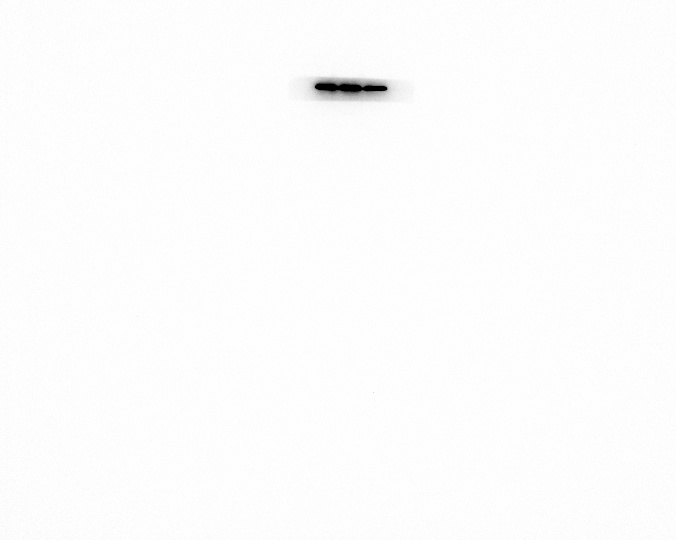
**
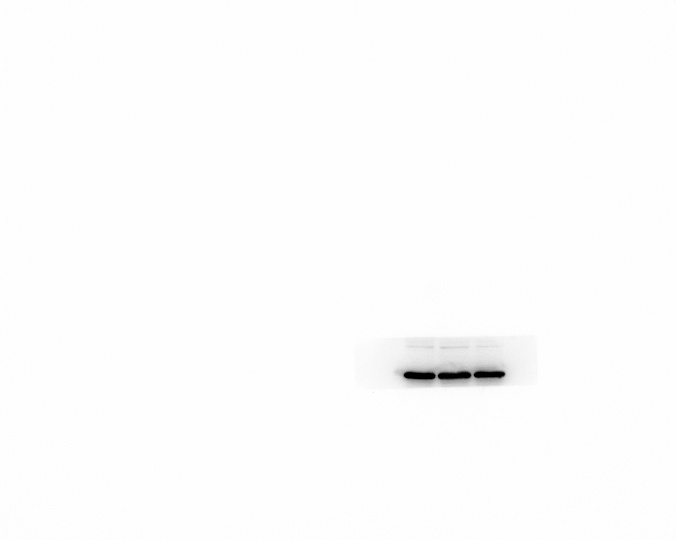


Cleaved casp1

Caspase1

control si-NC si-TRAF3

control si-NC si-TRAF3

Repeat4

Repeat3

55

Caspase1 45、42kDa

25

Cleaved casp1 12、10kDa

35

GAPDH 37kDa

control si-NC si-TRAF3

control si-NC si-TRAF3

GAPDH 37kDa

35

**Fig.5**

**
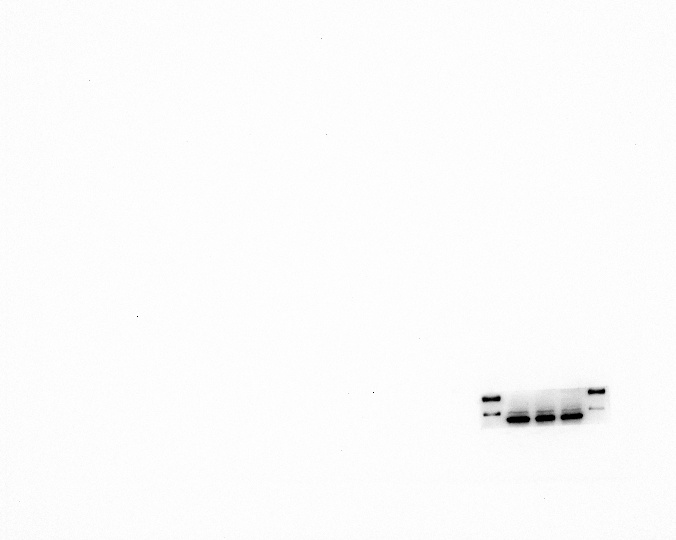

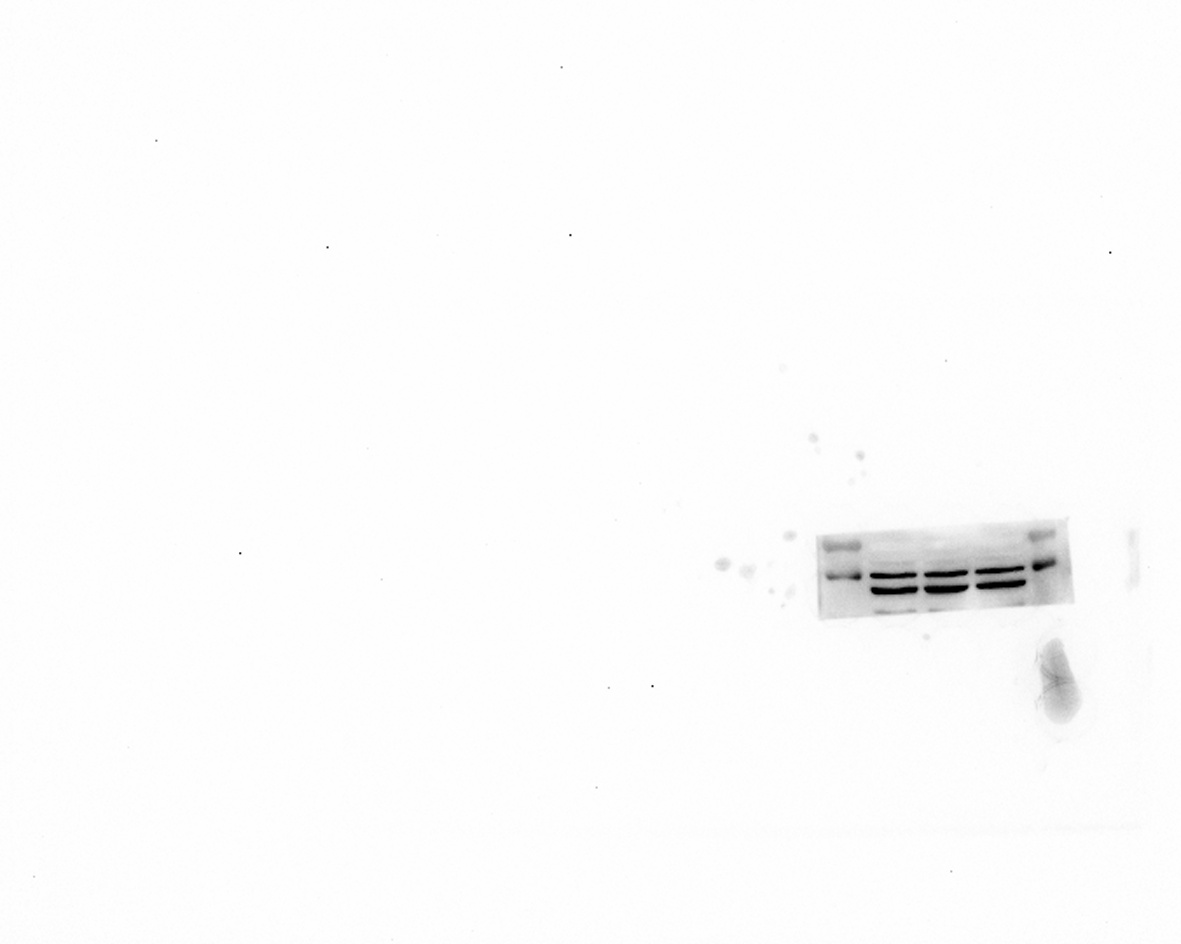
H1299**

GAPDH 37kDa

35

Cleaved casp1 12、10kDa

25

Caspase1 45、42kDa

55

Repeat1

Repeat2

Caspase1 45、42kDa

55

Repeat3

**
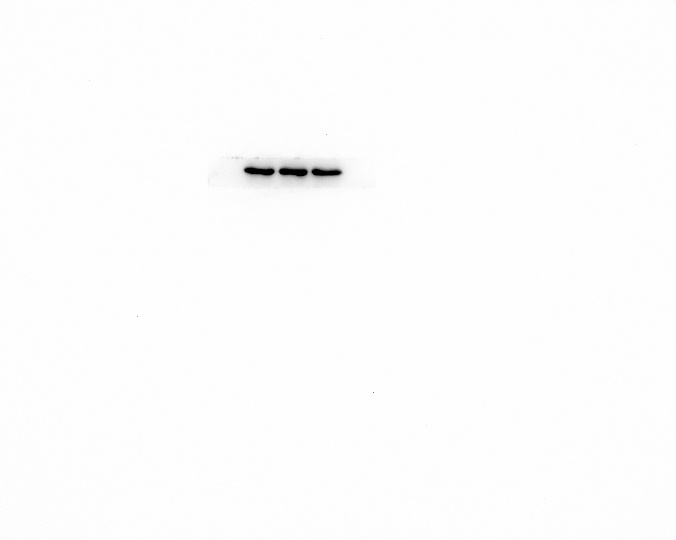

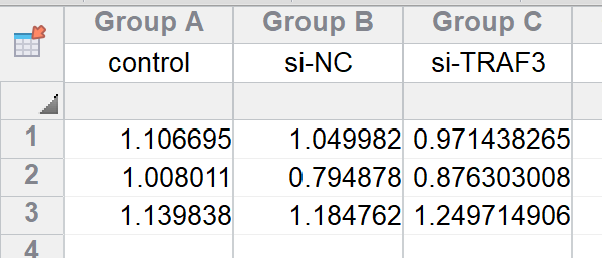

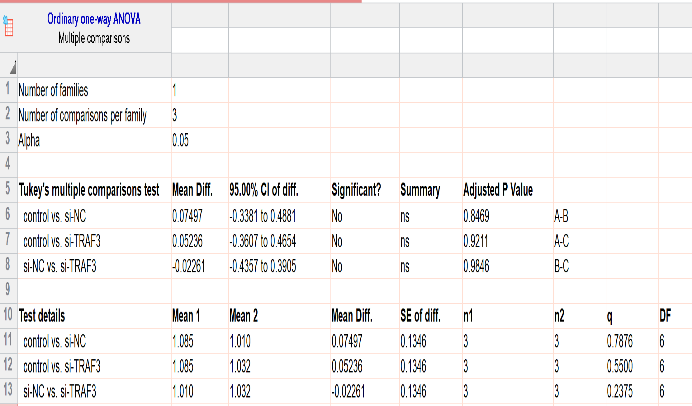

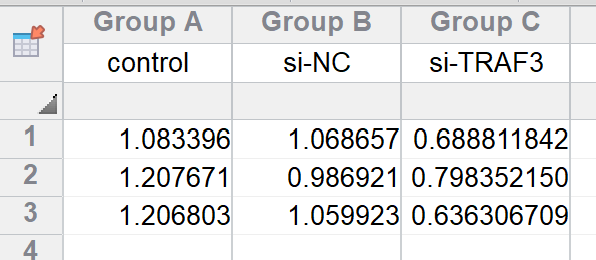

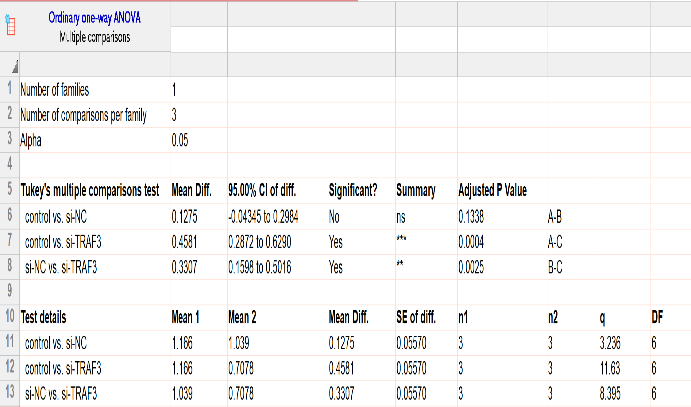

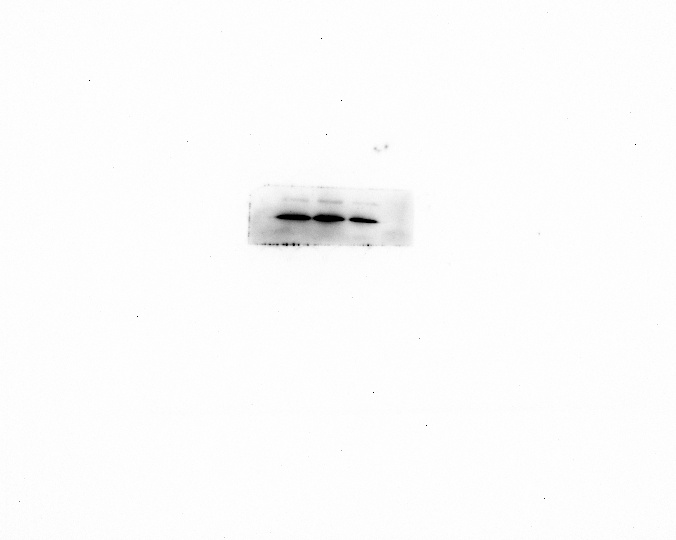

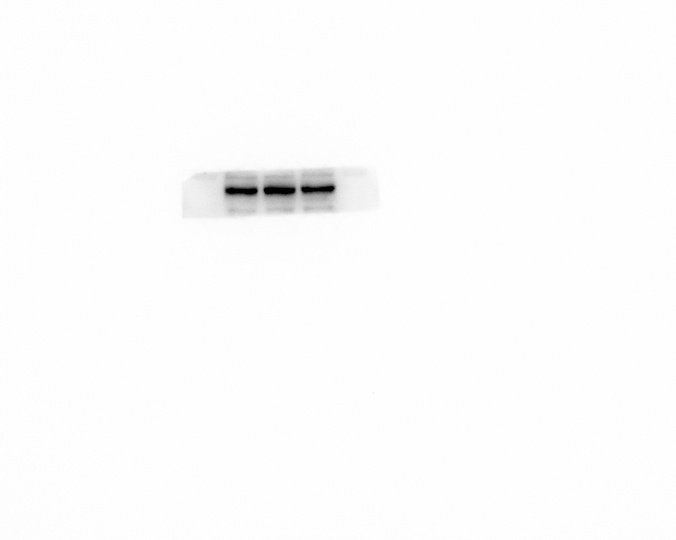

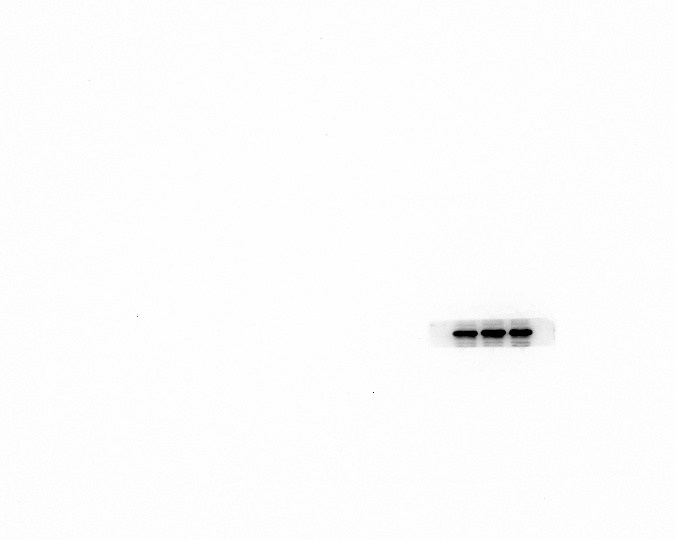

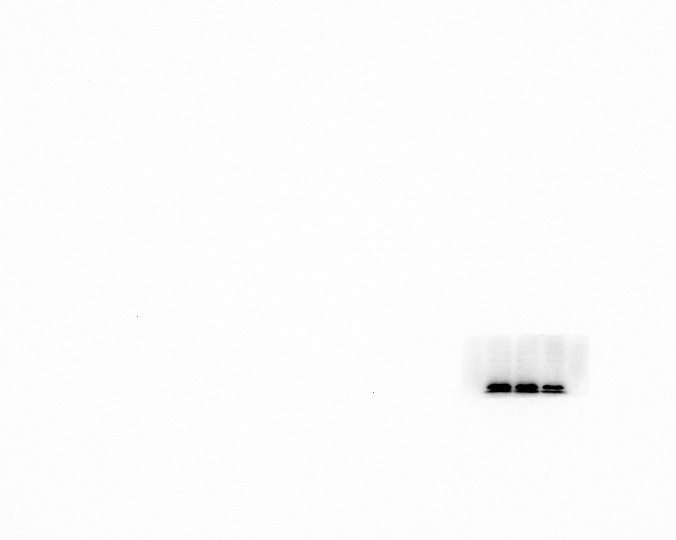

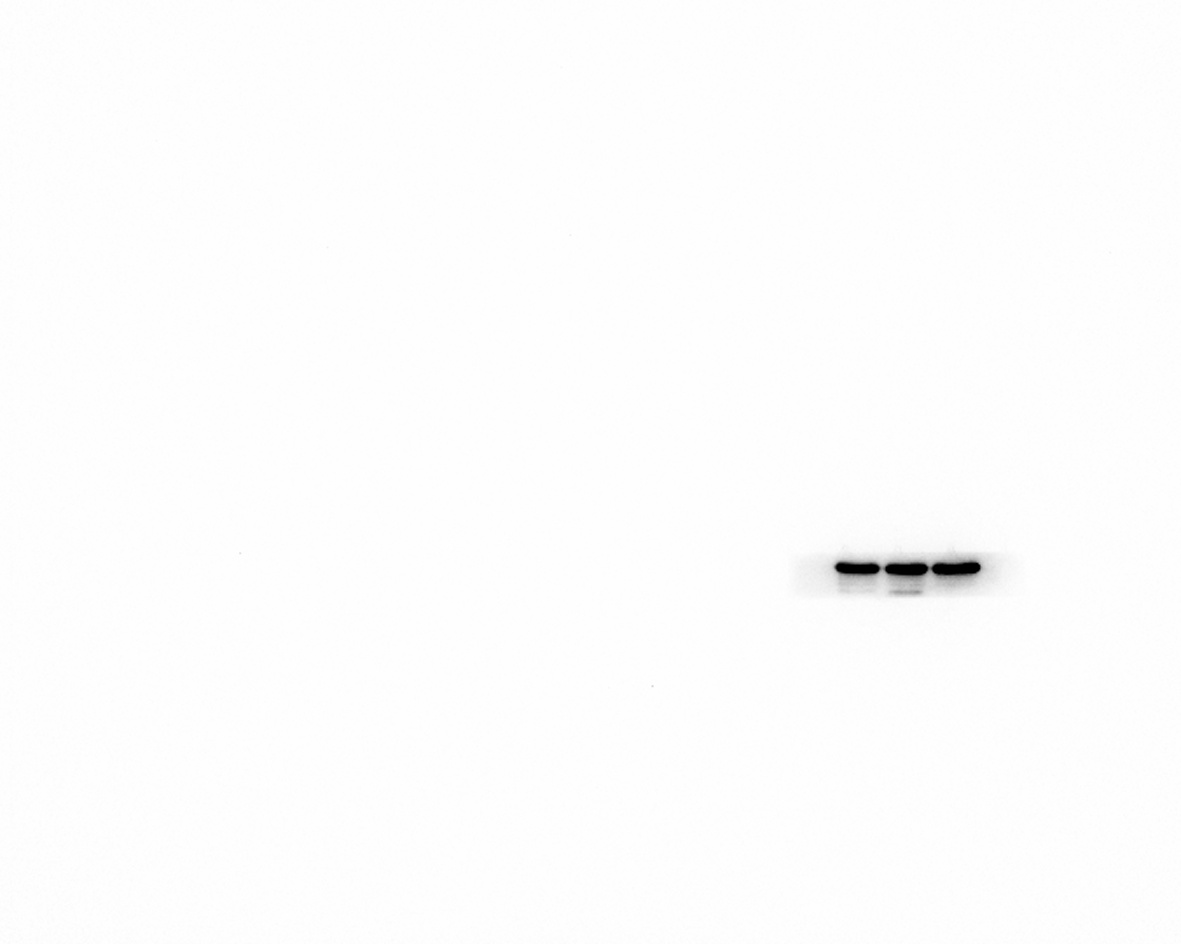

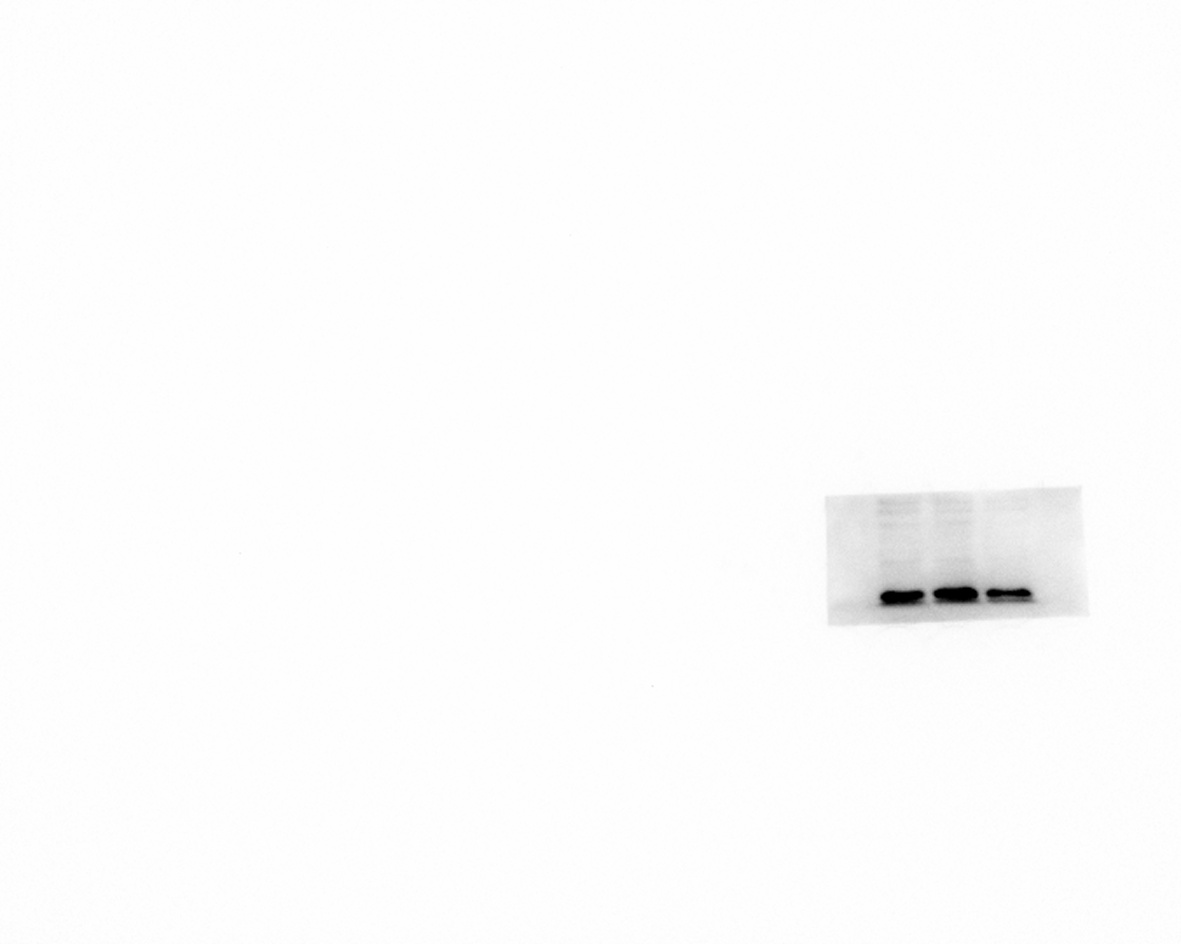
**

Caspase1

Cleaved casp1

control si-NC si-TRAF3

control si-NC si-TRAF3

control si-NC si-TRAF3

25

Cleaved casp1 12、10kDa

35

GAPDH 37kDa

**Fig.5**

Repeat1

Repeat2

Repeat3

Repeat4


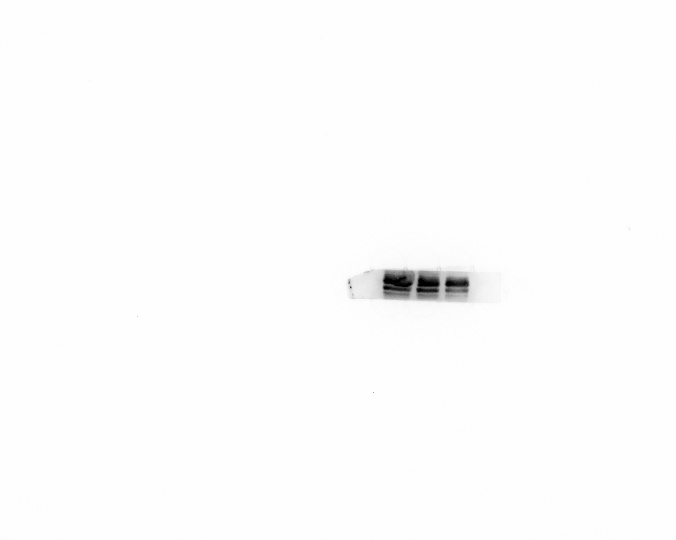
**
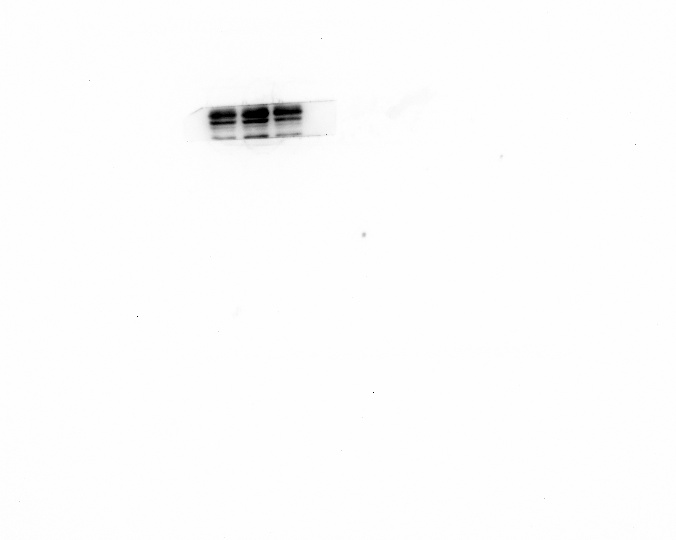
A549**

IL-1β 31kDa

**
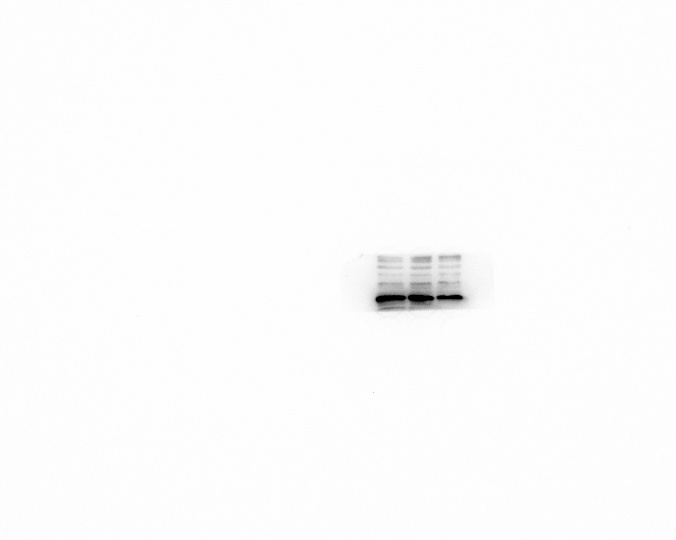
**
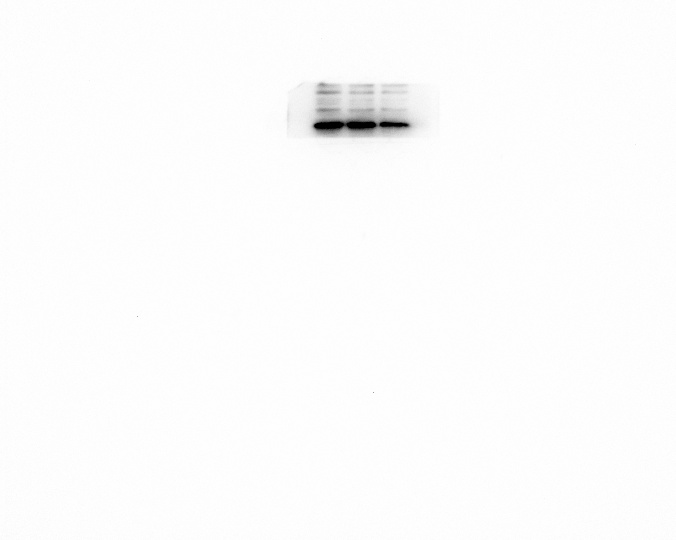
**
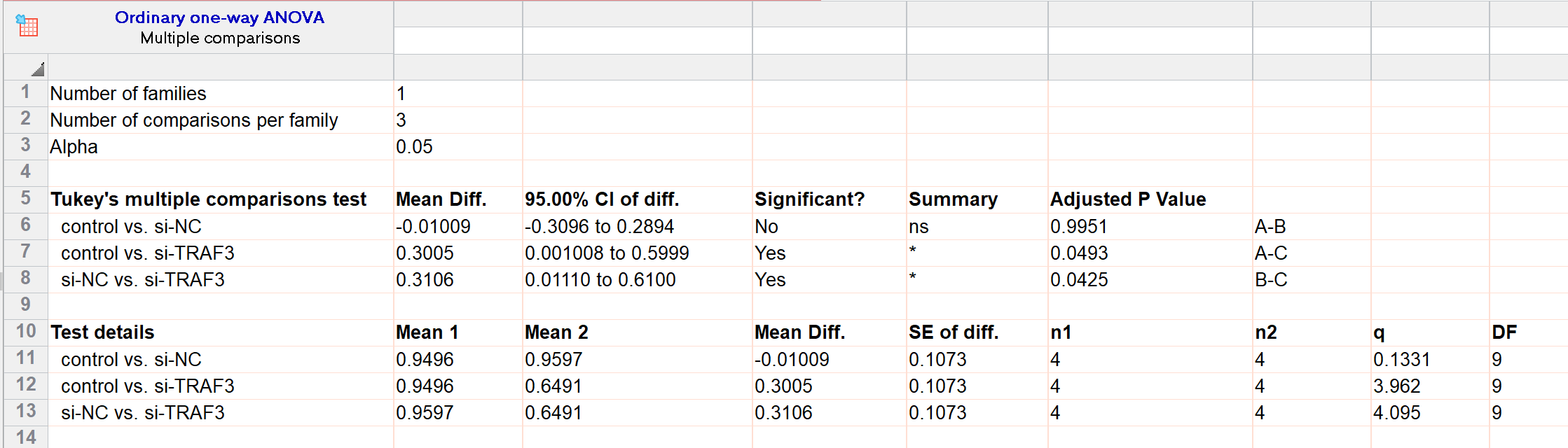

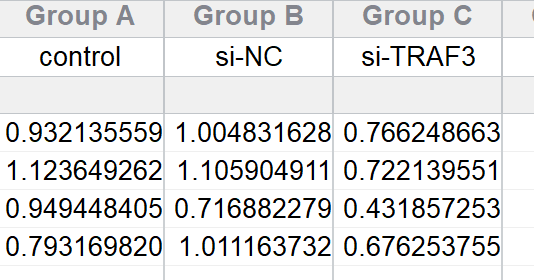

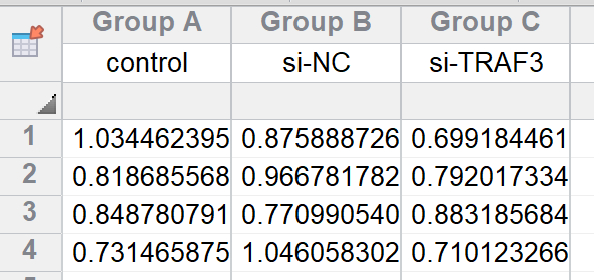

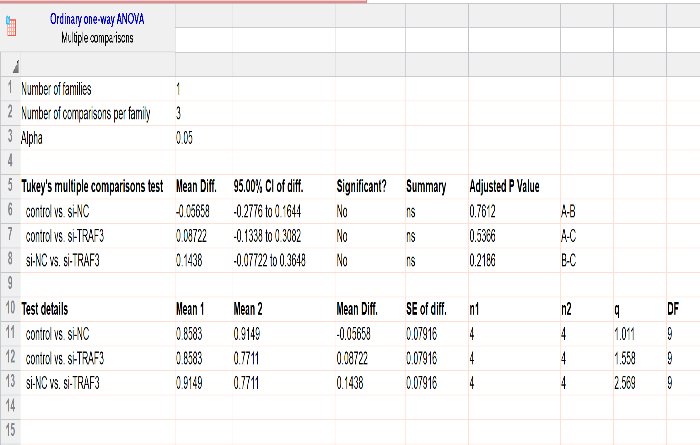

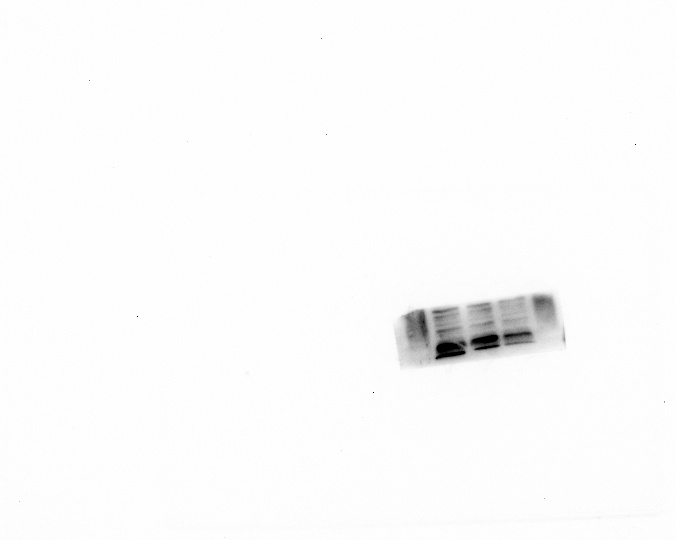

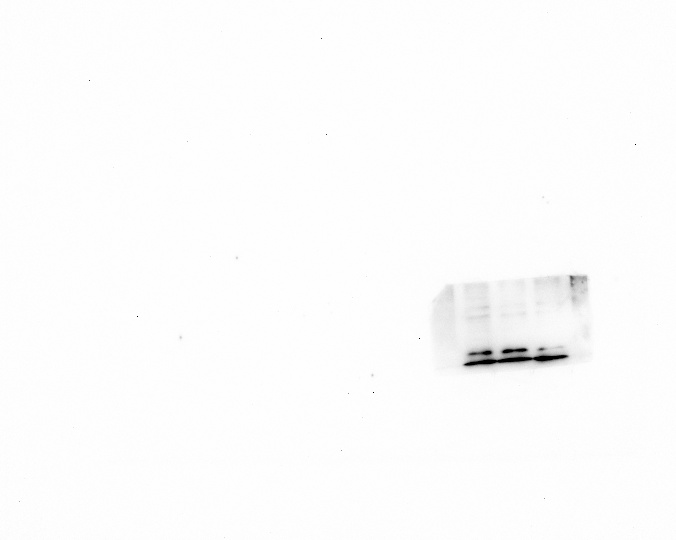

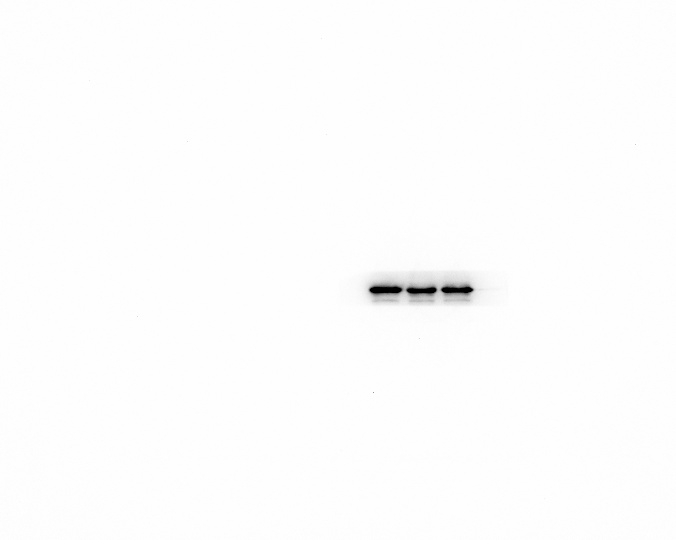

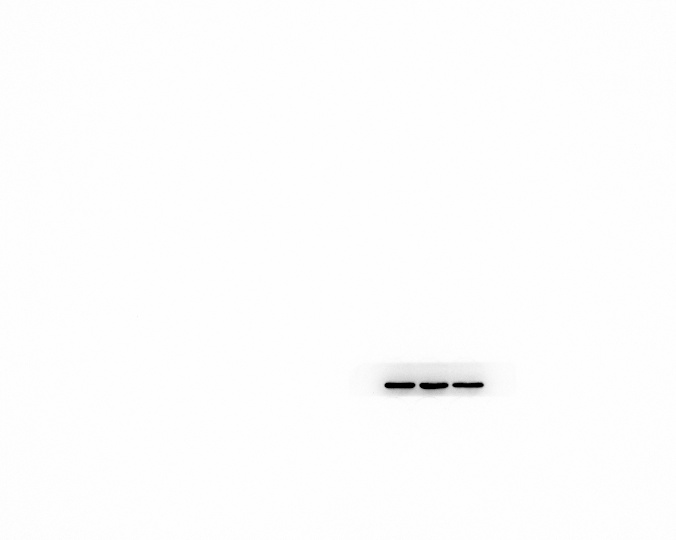

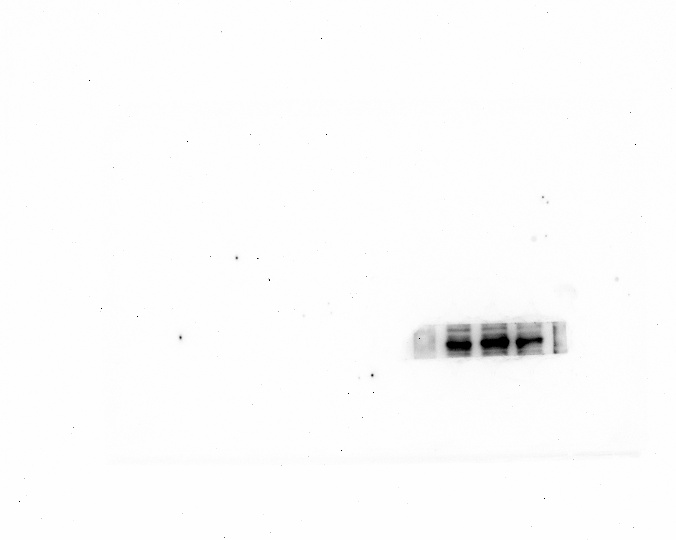

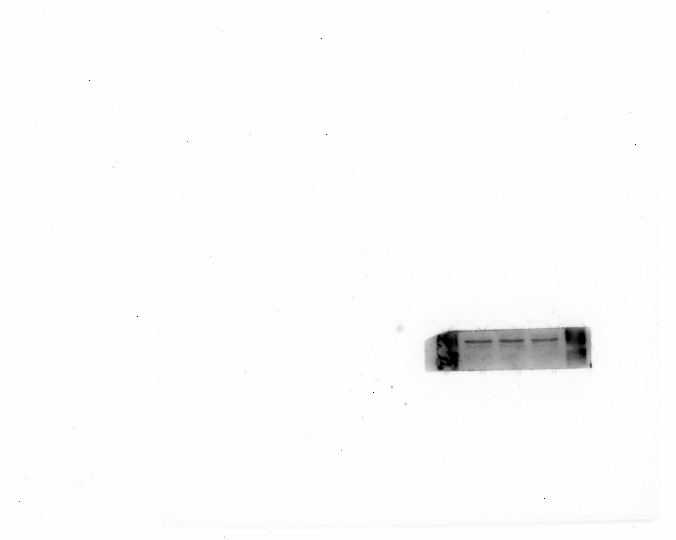

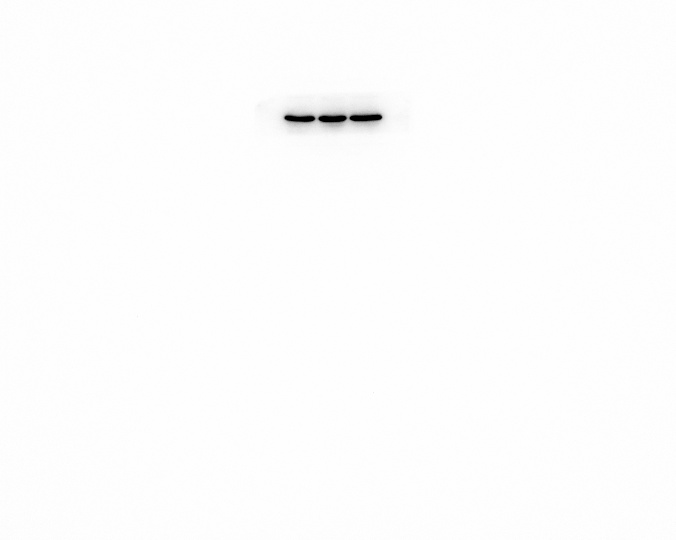

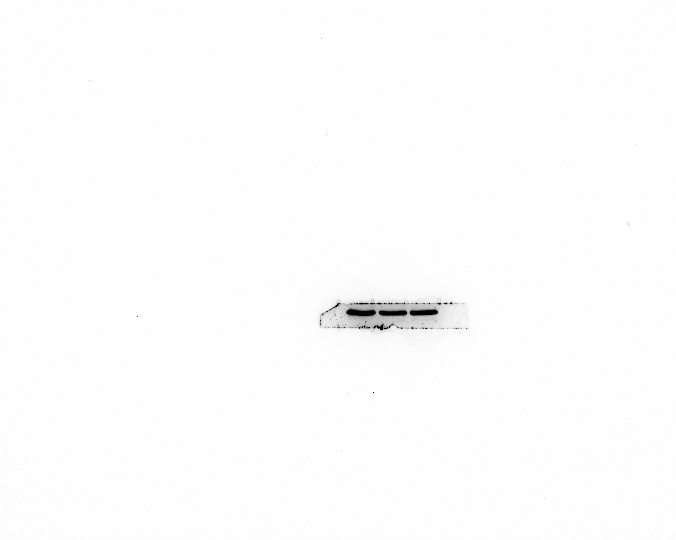
**

25

25

Mature IL-1β

IL-1β

Mature IL-1β 17kDa

IL-1β 31kDa

35

GAPDH 37kDa

Mature IL-1β 17kDa

35

GAPDH 37kDa

control si-NC si-TRAF3

control si-NC si-TRAF3

control si-NC si-TRAF3

control si-NC si-TRAF3

**Fig.5**

Repeat1

**
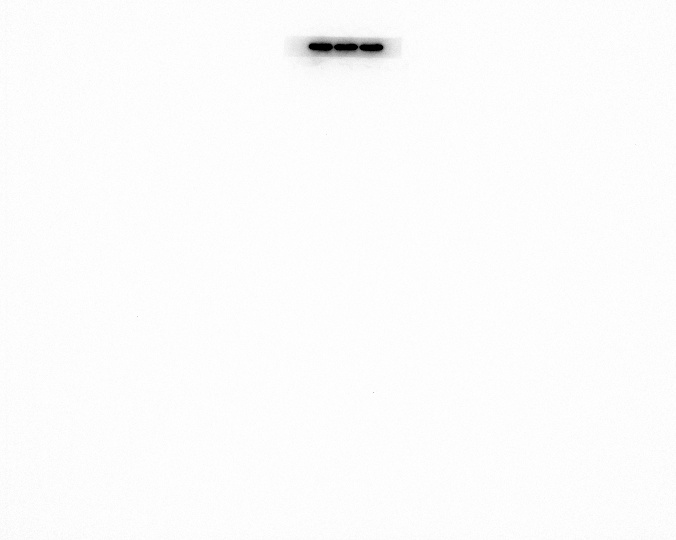

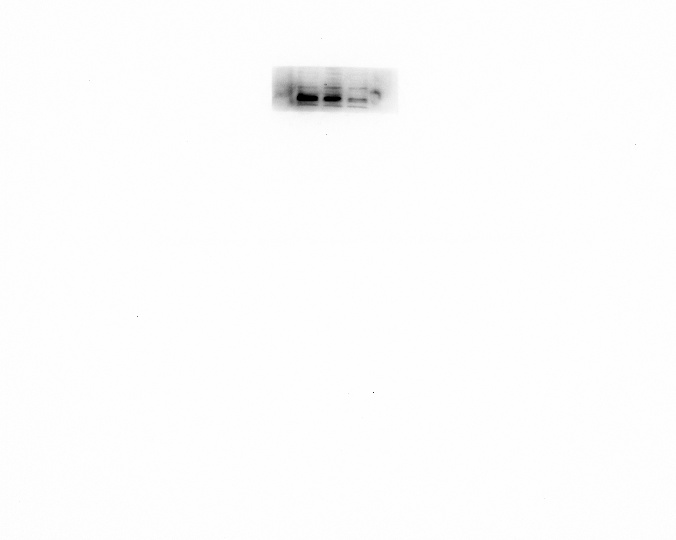

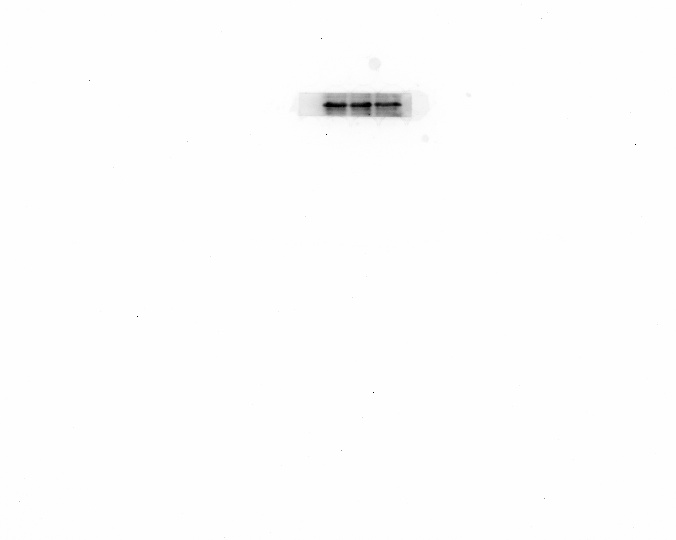
H1299**

Mature IL-1β

IL-1β

control si-NC si-TRAF3

control si-NC si-TRAF3

25

control si-NC si-TRAF3

control si-NC si-TRAF3

35

25

Repeat3

Mature IL-1β 17kDa

IL-1β 31kDa

35

GAPDH 37kDa

Repeat4

Repeat2

GAPDH 37kDa

IL-1β 31kDa

Mature IL-1β 17kDa
